# Deep residual networks for crystallography trained on synthetic data

**DOI:** 10.1107/S2059798323010586

**Published:** 2024-01-01

**Authors:** Derek Mendez, James M. Holton, Artem Y. Lyubimov, Sabine Hollatz, Irimpan I. Mathews, Aleksander Cichosz, Vardan Martirosyan, Teo Zeng, Ryan Stofer, Ruobin Liu, Jinhu Song, Scott McPhillips, Mike Soltis, Aina E. Cohen

**Affiliations:** a Stanford Synchrotron Radiation Lightsource, SLAC National Accelerator Laboratory, Menlo Park, CA 94025, USA; b Molecular Biophysics and Integrated Bioimaging Division, Lawrence Berkeley National Laboratory, Berkeley, CA 94720, USA; c Department of Biochemistry and Biophysics, UC San Francisco, San Francisco, CA 94158, USA; d Department of Statistics and Applied Probability, UC Santa Barbara, Santa Barbara, CA 93106, USA; e Department of Mathematics, UC Santa Barbara, Santa Barbara, CA 93106, USA; STFC Rutherford Appleton Laboratory, United Kingdom

**Keywords:** artificial intelligence, serial crystallography, rotation crystallography, synchrotrons, XFELs

## Abstract

The use of artificial intelligence to process diffraction images is challenged by the need to assemble large and precisely designed training data sets. To address this, a codebase called *Resonet* was developed for synthesizing diffraction data and training residual neural networks on these data. Here, two per-pattern capabilities of *Resonet* are demonstrated: (i) interpretation of crystal resolution and (ii) identification of overlapping lattices. *Resonet* was tested across a compilation of diffraction images from synchrotron experiments and X-ray free-electron laser experiments. Crucially, these models readily execute on graphics processing units and can thus significantly outperform conventional algorithms. While *Resonet* is currently utilized to provide real-time feedback for macromolecular crystallography users at the Stanford Synchrotron Radiation Lightsource, its simple Python-based interface makes it easy to embed in other processing frameworks. This work highlights the utility of physics-based simulation for training deep neural networks and lays the groundwork for the development of additional models to enhance diffraction collection and analysis.

## Abstract 2

Artificial intelligence was used to characterize the diffraction in images from serial and rotation crystallography experiments. Forward simulations were used to train models to infer *B* factors, resolutions and the presence of crystal splitting from single diffraction images.

## 1. Introduction

Crystallography data rates are on the increase at synchrotrons (SRs) and X-ray free-electron lasers (XFELs) alike. At SRs, high-brilliance undulator beamlines coupled with advances in robotics and detector technologies have accelerated the pace of experiments, requiring faster algorithms to provide feedback on experimental outcomes. For example, at the microfocus beamline 12-1 of the Stanford Synchrotron Radiation Lightsource (SSRL), data sets may be collected with crystal rotation speeds up to 90° per second and frame rates exceeding 100 Hz (Cohen, 2021 ▸). Beyond synchrotrons, XFEL facilities produce ultrashort and ultrabright X-ray pulses, making it possible to rapidly acquire high-resolution diffraction images with minimal radiation damage (Neutze *et al.*, 2000 ▸; Chapman *et al.*, 2011 ▸). At the Linac Coherent Light Source (LCLS), hard X-ray pulses are produced at 120 Hz, with similar rates reported at SACLA (Nango *et al.*, 2019 ▸), PAL (Park *et al.*, 2016 ▸) and SwissFEL (Milne *et al.*, 2017 ▸). At the European XFEL, using superconducting radiofrequency cavities (Singer *et al.*, 2015 ▸), hard X-ray pulses can be produced at 27 kHz (Weidorn *et al.*, 2018 ▸). Using similar technology, the up-and-coming LCLS-II facility is aiming to exceed this (Antipov *et al.*, 2018 ▸; Raubenheimer, 2018 ▸). While high-resolution diffraction images cannot currently be collected this fast, at XFELs the AGIPD (Allahgholi *et al.*, 2019 ▸) and JUNGFRAU 16M (Leonarski *et al.*, 2018 ▸) can record megapixel diffraction images at 3.5 and 1.1 kHz, respectively, and at SRs the Dectris EIGER can record at 0.8–3 kHz, depending on the model (Casanas *et al.*, 2016 ▸). A driving force behind these engineering advances is time-resolved protein crystallography, where biochemical reactions are initiated *in crystallo* and atomic-scale motions of proteins are mapped out by collecting multiple data sets along reaction timelines (Gruhl *et al.*, 2023 ▸; Schulz *et al.*, 2022 ▸; de Wijn *et al.*, 2022 ▸; Brändén & Neutze, 2021 ▸; Pearson & Mehrabi, 2020 ▸; Nango *et al.*, 2019 ▸; Pandey *et al.*, 2020 ▸; Šrajer & Schmidt, 2017 ▸; Schmidt, 2015 ▸). As this technology progresses, the use of automated processing tools will increasingly become necessary to improve beamtime efficiency and to optimize sample usage.

Work towards this goal has already progressed. For example, in Ke *et al.* (2018 ▸) the authors trained a convolutional neural network to determine whether an image contained any sign of diffraction from protein crystals. This neural network could then hypothetically be used to distinguish ‘hits’ from so-called ‘misses’, *i.e.* images with/without diffraction. These ‘misses’ (which comprise significant percentages of data collected using high-flow-rate injector methods) could then be excluded from processing and/or recording to disk to free up computing resources. More recently, in Rahmani *et al.* (2023 ▸) various dimensionality-reduction algorithms have been used to convert diffraction data into a set of features suitable for training a machine-learning classifier to automatically detect whether experimental images contained diffraction.

The above methods could be useful in scenarios where a large fraction of images contain misses (for example liquid-injection experiments). However, their utility is limited in cases of high-frame-rate experiments where most or all of the collected images contain diffraction, for example fixed-target serial crystallography (Lieske *et al.*, 2019 ▸; Baxter *et al.*, 2016 ▸; Cohen *et al.*, 2014 ▸) and high-speed rotational crystallography (Cohen, 2021 ▸). We propose here to move beyond the binary detection of diffraction and to use artificial intelligence (AI) to describe the observed crystal diffraction with quality-indicating metrics. For the presented work, an AI was trained to answer the following questions: (i) ‘What is the crystal resolution?’ and (ii) ‘Is there parasitic diffraction from overlapping lattices?’. Crystallographers can readily find answers to these questions using visual inspection, but this practice is inefficient and impractical at high data rates. Conventional crystallo­graphic algorithms can answer the first question but are sensitive to input parameters and image artifacts. For example, the resolution-estimation program implemented in the *DIALS* software suite (Winter *et al.*, 2018 ▸) is sensitive to image artifacts from ice diffraction and pixels that record high values arising from unknown, external sources (so-called ‘hot pixels’). The *CrystFEL* suite also provides per-shot resolution estimates for stills using indexed reflections (White *et al.*, 2016 ▸); however, the results are sensitive to the indexing parameters. Regarding the second question, diffraction from overlapping lattices (where Bragg peaks from multiple crystals are not well separated) can hinder data processing. Depending on the degree of peak separation, partially overlapping lattices are exceedingly difficult to detect using conventional methods, with detection usually requiring specialized indexing capabilities (see, for example, Gildea *et al.*, 2014 ▸; Schmidt, 2014 ▸).

To answer the above questions with AI models, forward-simulation software was used to create vast and diverse training data sets of X-ray diffraction images. Specific aspects of diffraction were thus minutely controlled. Crucially, the synthetic images were automatically labeled according to the underlying physics. The *PyTorch* library was then used to train a regression model for resolution prediction and a classification model to label overlapping lattice diffraction. Both models accepted a two-dimensional diffraction pattern as input, after applying a simple downsampling filter.

The trained *Resonet* models were tested using previously collected data representing a wide variety of detectors and sources. *Resonet* models were also tested during live data collection at several SSRL crystallography beamlines. Because they have no tunable parameters, *Resonet* models were found to be well suited for automated diffraction monitoring (see Section A3). During rotation data collection, inferences from *Resonet* models can also be used to monitor for radiation damage, crystal mis-centering and asymmetric diffraction. During serial experiments on BL12-1 at SSRL, *Resonet* results can be used to optimize experimental parameters such as injector flow rate, X-ray attenuation and/or beam size. Other diffraction-monitoring applications can easily use *Resonet*, especially Python-based programs such as *OM* (Mariani *et al.*, 2016 ▸). Work to expand *Resonet* to predict even more parameters of interest is ongoing, driven by a goal to produce a stable, high-performance framework for general use at crystallography facilities worldwide.

## 2. Methods

### 2.1. Simulating training data

To generate training data from which to build prediction models, we used *nanoBragg* (Holton *et al.*, 2014 ▸; Lyubimov *et al.*, 2016 ▸; Sauter *et al.*, 2020 ▸), which simulates X-ray diffraction by macromolecular crystals according to the kinematic theory of diffraction (James, 1962 ▸). The *nanoBragg* program incorporates user-defined background scattering and adds noise by sampling Poissonian and Gaussian distributions describing photon counting and electronic noise, respectively. The use of simulated images facilitates the creation of large training data sets that would be impractical to accurately sort and label by hand. Furthermore, it becomes possible to create training data sets that vary or isolate any combination of properties. For all of the simulations reported here, a variety of parameters were randomly sampled, including detector distances, detector types, beam-stop sizes, bad-pixel masks, hot-pixel masks, proteins, space groups, unit cells, crystal volumes, mosaic spreads and background scatter. These are summarized in Appendix *A* [App appa], Sections A1.1–A1.5 [Sec seca1.2] [Sec seca1.3] [Sec seca1.4] [Sec seca1.5]. For each simulated image, only one quadrant was used for training (the upper left) and stored as a maxpool-downsampled array of 512 × 512 pixels.

#### 2.1.1. Resolution training data

Resolution is perhaps the most important quality metric in any structural biology experiment because it defines the clarity of the structural image. Formally, resolution is the minimum separation distance between two features required for these two features to be identified as distinct from one another, for example at 1 Å resolution individual atoms can be clearly resolved, whereas at poorer resolutions (2–3 Å) amino-acid side chains are resolvable but individual atomic positions must be inferred from prior knowledge and are less reliable. In practice, X-ray crystallographers determine the resolution cutoff as the point at which the merged diffraction data become uninterpretable. Criteria for inferring resolution have evolved over the decades. Oftentimes, the recent and widely accepted CC_1/2_ metric defined by Karplus & Diederichs (2012 ▸) is used to set the resolution cutoff, while in other cases the related signal-to-noise ratio of the structure-factor intensities is used. In this work, we used the latter approach to define a resolution for comparison with *Resonet* inferences.

**Figure 1:**
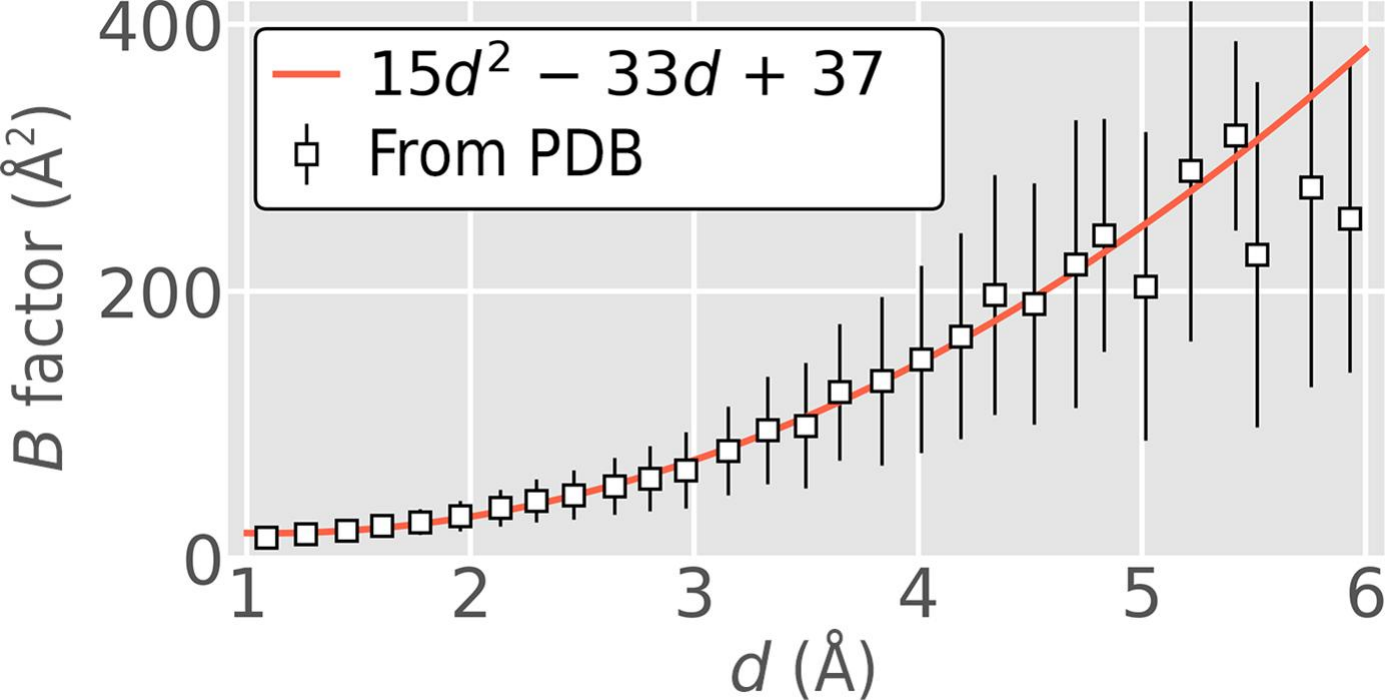
Resolution versus *B* factor. Structures from the PDB indicate an overall trend in resolution versus *B* factor, as determined by a simple quadratic fit (red line). The square markers shown here represent average *B* factors at each resolution across the entire PDB, and the error bar is one standard deviation. The fit was only performed using data in the interval 1–4.5 Å. This trend becomes unreliable at resolutions of >5.5 Å.

The ‘resolution of a diffraction pattern’ is also a concept that is commonly used when discussing X-ray diffraction experiments themselves and is defined by the widest angle from the incident beam at which Bragg peaks can be observed. Observability of the Bragg peaks is in turn related to the rate at which the diffraction decays on the image, parameterized by a quantity called the *B* factor (Bragg, 1914 ▸). Higher *B* factors indicate disorder in the crystal due to uncertainties in atomic positions arising from thermal motions, which ultimately affect the resolution of a data set, causing diffraction to fall off more rapidly with resolution and obscuring reflections at wider scattering angles. *B* factors and resolutions are included with structures deposited in the Protein Data Bank (PDB; Berman *et al.*, 2000 ▸), which makes them amenable to data mining. Thus, an analysis of *B* factors and resolutions revealed a simple nonlinear relationship that was first described in Holton (2009 ▸). This trend was updated to account for the more than a decade’s worth of new PDB structures since then (Fig. 1 ▸).

**Figure 2:**
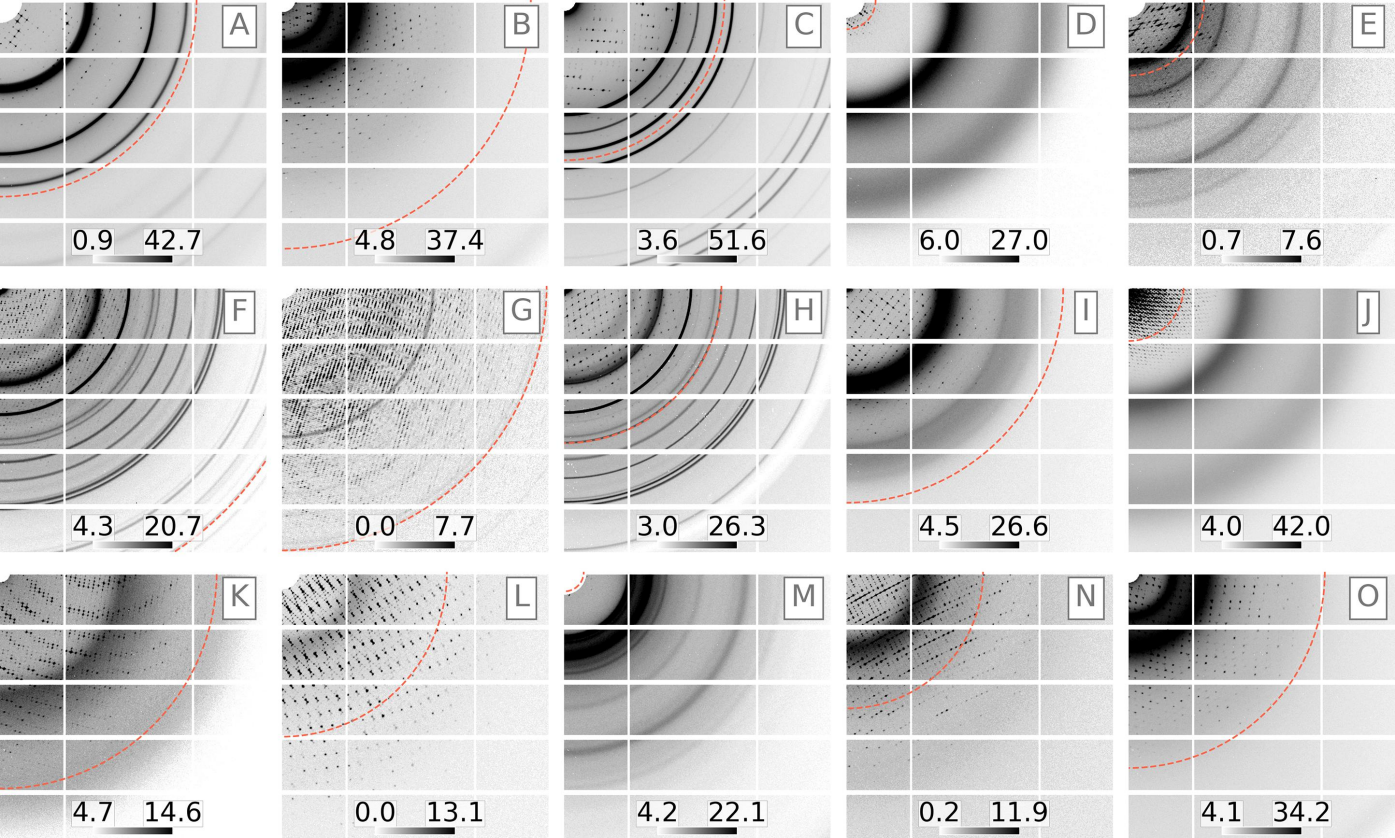
Simulated PILATUS 6M images with varying resolutions. Some parameters underlying each image are summarized in Table 1 ▸. For each sub-image, the beam center is in the upper left corner and the resolution (determined from the *B* factor) is indicated by a red dashed line. The sub-images represent one quadrant of a PILATUS 6M camera, downsampled to a 512 × 512 pixel array according to Section 2.2 (see also Fig. 4 ▸). Note that resolution here is related to the *B* factor by the relationship shown in Fig. 1 ▸. Hence, while the resolution sometimes appears intuitively as the point where the scattering drops off (for example in A, B, C, D, G, H, I, K and O), at other times the Bragg reflections extend to wider angles beyond the indicated resolution (E, J, L and N). This results from inaccuracy in the resolution-versus-*B* factor relationship (Fig. 1 ▸). Rarely, resolution is obscured by large background (F) or is covered by the beam stop (M). These edge cases add noise to the model training. Color bars are shown in square-root-photon units.

With this relationship between *B* factor and resolution as an underlying assumption, a resolution-prediction training data set was created by simulating images with varying *B* factors. Fig. 2 ▸ shows a randomly selected assortment of these simulated images and their corresponding resolutions. Some parameters underlying each image are summarized in Table 1 ▸. Note that the resolution cutoff does not always align with the point at which the diffraction becomes invisible in the image. Instead, resolution is defined here by the rate of diffraction intensity decay as expressed by the *B* factor. However, it is complicated by the varying degrees of background in each image: a high-resolution image can also have a high background that makes it appear to be a low-resolution image (Fig. 2 ▸ *f*), adding uncertainty to our training data labels. Further, specific to synchrotron experiments, the dose received by a crystal also influences the *B* factor (Holton, 2009 ▸; Kmetko *et al.*, 2006 ▸) and ultimately the resolution. A strategy to account for these additional factors is described in Holton & Frankel (2010 ▸), but for the main results presented here we rely on the generality of the relationship between *B* factor and resolution shown in Fig. 1 ▸ and note that the *B* factor is the dominant term affecting the damage-limited intensity from a protein crystal, appearing as a Gaussian expression in equation (18) in Holton & Frankel (2010 ▸). Resolution training data were simulated on a combination of PILATUS 6M and EIGER 16M camera models with variable detector distances in the range 200–300 mm. All simulations assumed a fixed photon energy of 0.9795 Å. See Sections A1.1–A1.5 [Sec seca1.2] [Sec seca1.3] [Sec seca1.4] [Sec seca1.5] for further details.

#### 2.1.2. Overlapping lattice training data

**Figure 3:**
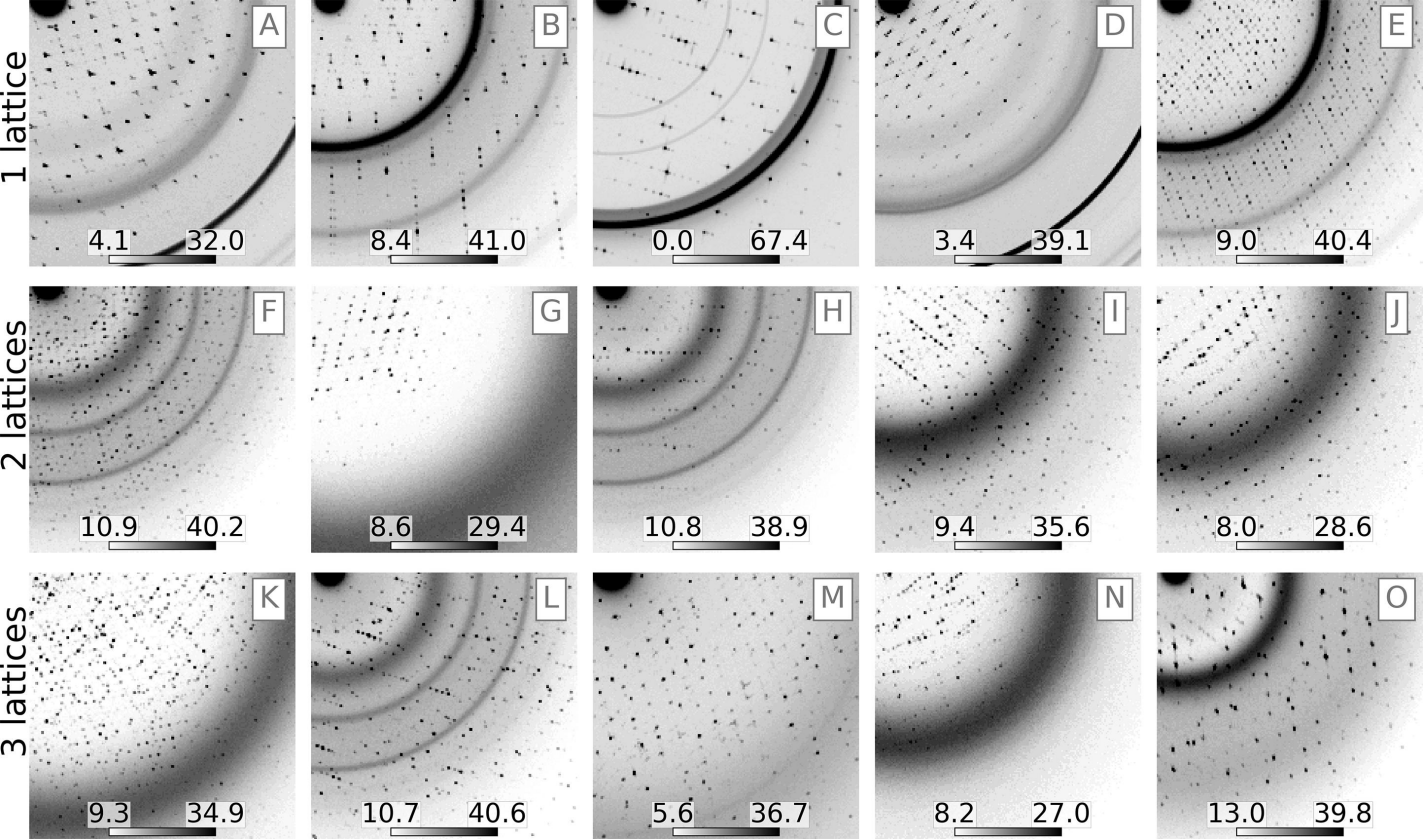
Simulated diffraction from one lattice (top row), two lattices (middle row) or three lattices (bottom row). Each sub-image represents the lower quadrant of a Rayonix camera, downsampled as illustrated in Fig. 4 ▸. Color bars are shown in square-root-photon units.

Overlapping lattice scattering occurs when multiple crystal domains are exposed simultaneously, either because the diffracting volume contains a crack or a major dislocation or if several crystals are caught in the beam. This effect undermines diffraction data-processing algorithms, which for the most part assume that diffraction comes from a single lattice. To simulate training data for overlapping lattice scattering, a random number of lattices (1, 2 or 3) were ‘placed’ in the simulated X-ray beam in randomized orientations. For this training, rotational mosaic spread was kept small (<0.01°) and overlapping lattice spacings were drawn from a Gaussian distribution with a randomly chosen variance of 0.1°, 1° or 10° and a mean of 0° (about the nominal crystal orientation). In this way, it was theoretically possible for Bragg peaks from different lattices to closely overlap in a single image, thus simulating diffraction from a cracked crystal. Fig. 3 ▸ shows a randomly selected assortment of overlapping lattice training data and illustrates how image features vary with the number of lattices. Training data for this model used a Rayonix 340 (Rayonix LLC) detector format matching the geometry from an LCLS experiment (Artz *et al.*, 2020 ▸); however, it was found that the model generalized well to other data sets using different detectors (as described in Section 3). The training data set was made up of 50% single-lattice images, 25% two-lattice images and 25% three-lattice images.

### 2.2. Image conditioning

**Figure 4:**
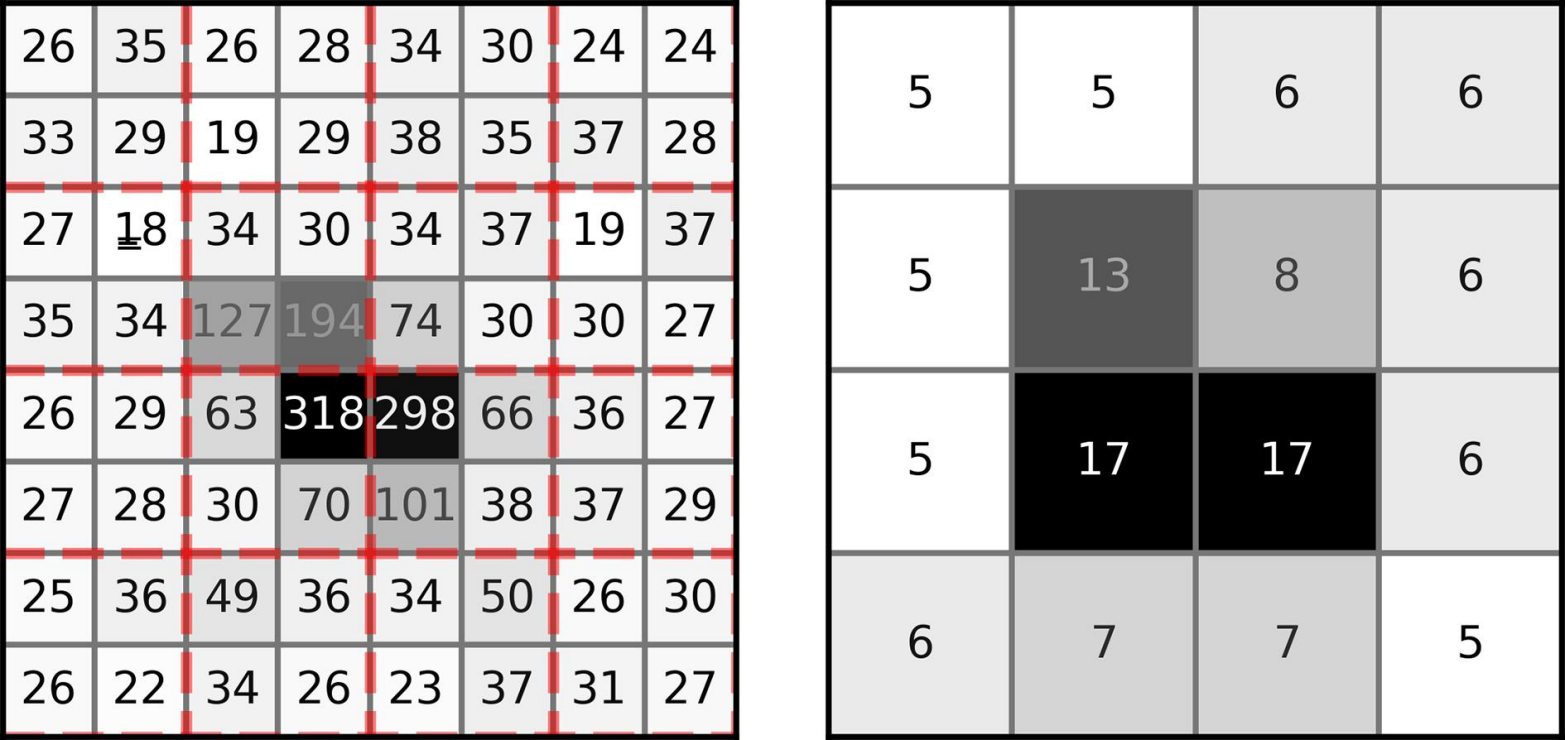
Downsampling scheme. A region of a PILATUS 6M image with a Bragg reflection is shown (with numbers indicating pixel values). Raw data (left) are divided into blocks of pixels (indicated by red dashed lines). This occurs for both simulated and experimental data. The ‘conditioned’ pixel value (right) is the square root of the maximum pixel value within each block, cast to an integer (floor operation). Block size varies according to the detector model; either 2 × 2 blocks (PILATUS 6M) or 4 × 4 blocks (EIGER 16M, JUNGFRAU 16M and Rayonix 340) were used.

All images (both simulated and experimental) were downsampled and normalized before model evaluation, as the raw data formats considered for this study (Dectris PILATUS 6M, Rayonix 340, JUNGFRAU 16M and Dectris EIGER 16M) are large. To downsample an image by a factor of *N* (*N* = 2 for PILATUS 6M; *N* = 4 for EIGER 16M, JUNGFRAU 16M and Rayonix), the raw pixels were grouped into *N* × *N* blocks and the value of each ‘block pixel’ was set as the maximum value of the *N* ^2^ raw pixels inside it. The downsampled ‘block pixel’ values were then replaced by their square root and cast as integers. This data-conditioning process is shown in detail in Fig. 4 ▸ for a region of a PILATUS 6M image containing a Bragg reflection. After downsampling, the images were divided into four quadrants of size 512 × 512 pixels, each of which could be passed to our AI-trained models to produce independent estimates for predictors. Preliminary tests revealed that the above downsampling and normalization scheme lead to better training when compared with simply averaging pixels together. Further testing is needed to determine whether more optimal preconditioning could lead to faster training and/or more accurate models.

### 2.3. Model fitting

*PyTorch* (Paszke *et al.*, 2017 ▸) was used to fit regression (resolution prediction) and classification (overlapping lattice detection) models using our training data sets. In general terms, *PyTorch* was tasked with reducing the error (‘loss’) between the ground-truth labels and those derived from the current model. For resolution-prediction training, the loss function was the mean squared error between labels and predictions in inverse units, *i.e.* inverse resolution was predicted by the model and compared with inverse-resolution labels (for example, an image simulated with a *B* factor corresponding to 2 Å resolution was labeled by 0.5 Å^−1^). For overlapping lattice-detection training, the binary cross-entropy loss function was used. Training labels were set to 0 or 1 (single lattice or overlapping lattices) and model predictions were mapped to a probability using a sigmoid function and then rounded to 0 or 1 before computing the loss.

#### 2.3.1. Model architecture

**Figure 5:**
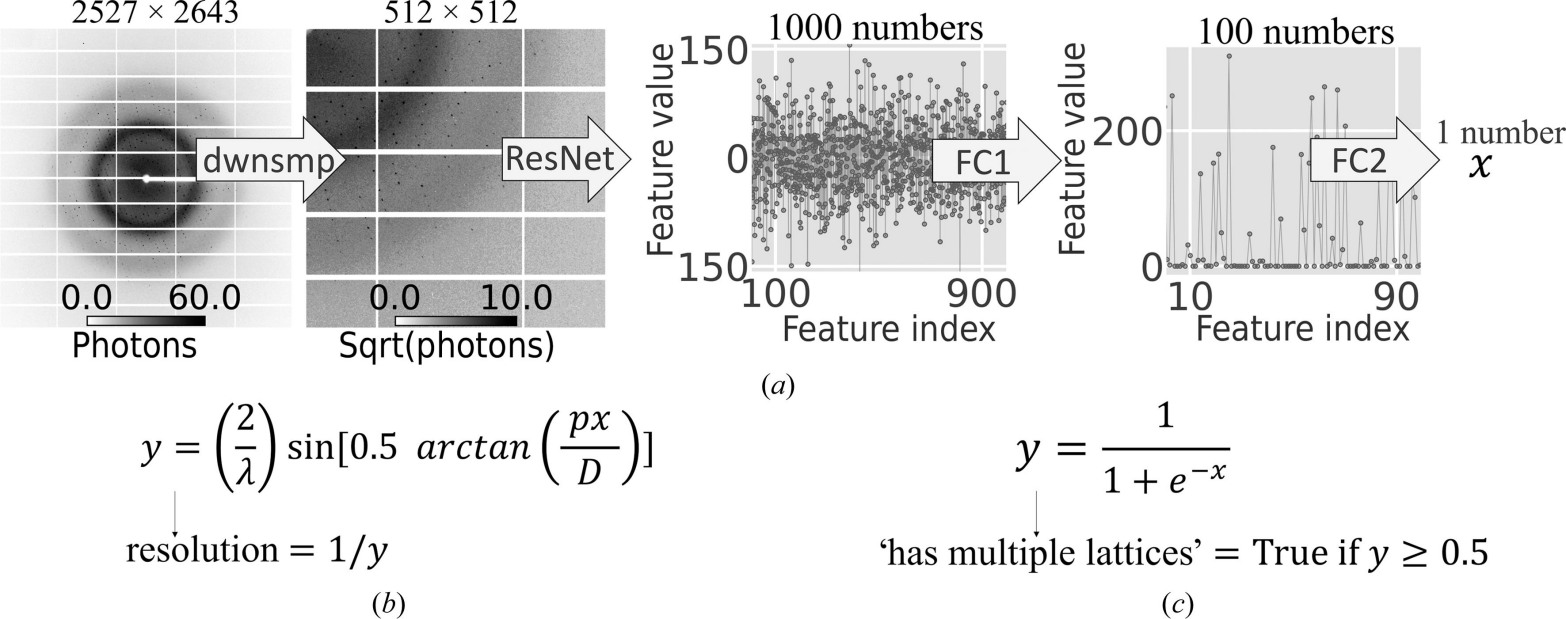
Model architecture. (*a*) Raw data were downsampled as described in Section 2.2, forming four 512 × 512 quadrants. Quadrants were then passed through a ResNet architecture, resulting in 1000 features. Next, a series of fully connected layers (FC1, FC2) was used to convert the 1000 features into a scalar value. If predicting resolution (*b*), this was converted to an inverse resolution using the diffraction wavelength (λ), downsampled pixel size (*p*) and sample-to-detector distance (*d*). If predicting overlapping lattice scattering (*c*), this scalar was passed through a sigmoid function and then rounded, such that 0 and 1 indicated single and overlapping lattice scattering, respectively. The image and line plots in (*a*) are from a real experimental image as it was passed through the fully trained resolution model. The inferred resolution in this case was 1.67 Å. Table 2 ▸ describes the number of parameters in the different model stages (ResNet, FC1, FC2). One quadrant was sufficient to predict the quantities of interest; however, repeated model passes with the second, third and fourth quadrants can provide a measure of uncertainty in the predicted values.

Currently, *Resonet* uses a residual network (ResNet; He *et al.*, 2015 ▸) architecture with a modified input/output stage for predicting resolution and detecting overlapping lattices. ResNet is a state-of-the-art deep convolutional neural network architecture which accepts RGB images as input. For each image, it outputs 1000 numbers (features) intended for use in a multi-class classification model (with up to 1000 possible outcomes). To use ResNet with diffraction images, its input layer (a convolutional layer) was modified to accept single-channel (greyscale) images. Secondly, as originally performed in Lecun *et al.* (1998 ▸), two fully connected (FC) layers were chained together at the output stage to convert the 1000 numbers into a single number suitable for prediction. The first FC layer mapped 1000 numbers to 100 numbers using 100 + 10^5^ parameters, while the second FC layer mapped 100 numbers to one number (using 1 + 10^2^ parameters). Also, following Lecun *et al.* (1998 ▸), a rectified linear unit activation function was used between the first and second FC layers (see Fig. 5 ▸), adding nonlinearity to the FC models. Fig. 5 ▸(*a*) shows the baseline architecture used for both resolution and overlapping lattice-prediction models. Each model has unique aspects related to the desired predictor. For resolution, an additional input vector of basic diffraction-geometry quantities (detector distance, pixel size and wavelength) was used to convert the output of the base model to an inverse-resolution quantity (Fig. 5 ▸ *b*). Modeling inverse resolution prevented scenarios where zero-division could occur during model training. For overlapping lattice detection, a sigmoid function was used to convert the output to the range 0–1, typical for binary classification (Fig. 5 ▸ *c*).

#### 2.3.2. Model training

**Figure 6:**
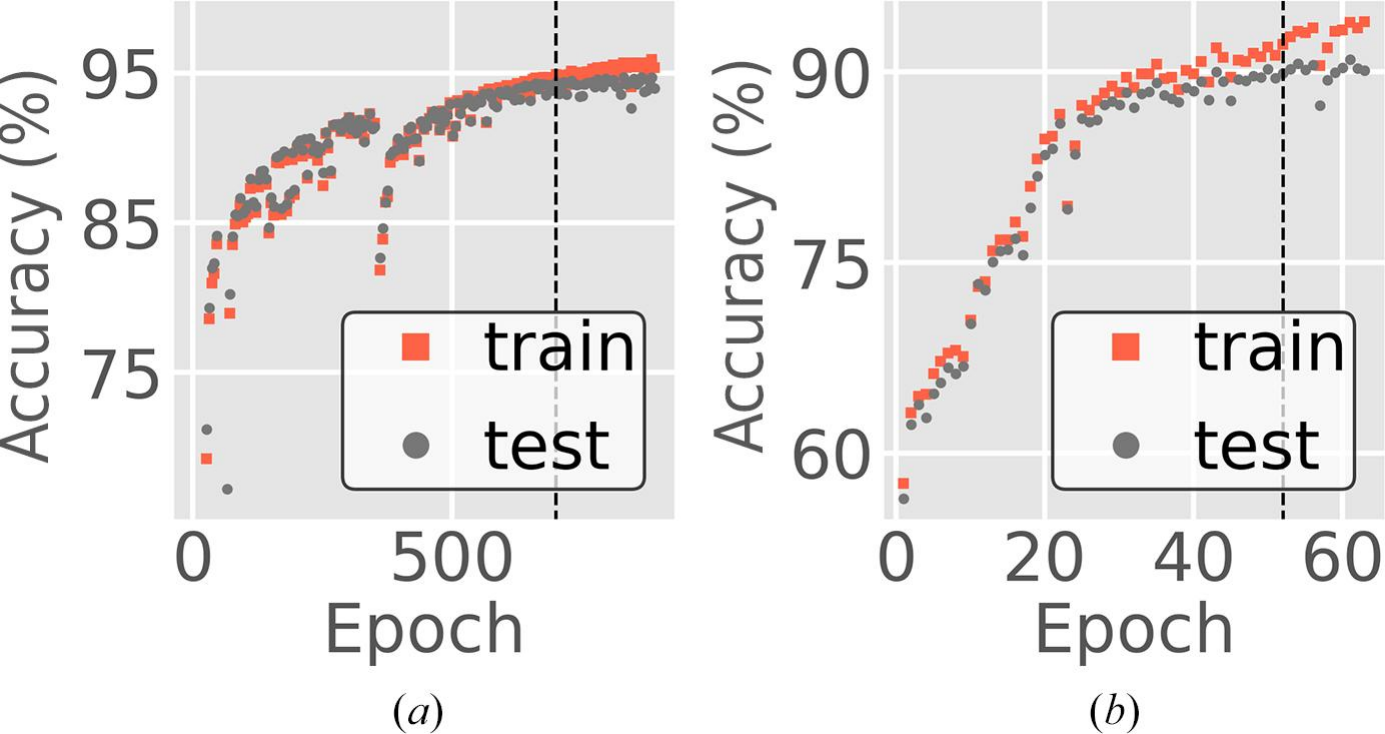
Training optimization curves. (*a*) Accuracy versus training epoch for the resolution-prediction model. This is a regression model, for which we define accuracy as the fraction of images whose predictions are within 0.07 Å^−1^ of the ground truth. The training job was stopped after epoch 354 and then restarted, as indicated by the discontinuity. (*b*) Accuracy versus epoch for the overlapping lattice-detection model. Here, accuracy is the fraction of predictions with the correct label. For both plots, the test curves (black markers) are derived from images that were never used for training. Eventually, training accuracy diverges, indicating model bias. The vertical lines mark the epoch where models were saved for use with experimental data.

For the resolution-prediction model, training was performed on a data set comprising 200 000 PILATUS 6M and 125 000 EIGER 16M images, each labeled with a unique resolution according to its *B* factor, and with a randomized sample-to-detector distance. After each epoch (a pass through the entire training set, computing the loss function and its gradient for every training example), the model was validated on 10% of the simulated images that were set aside for testing and not included in training. The resolution-inference training loss curve is shown in Fig. 6 ▸(*a*) for both the training and test sets. Training was carried out on 16 Perlmutter GPU nodes at NERSC, utilized 64 A100 GPUs and ran at a speed of 0.7 min per epoch. For the overlapping lattice-detection model, training was performed using 117 000 simulated diffraction images, each labeled by a Boolean indicating the presence of overlapping lattices, and at each epoch the model was validated on 13 000 simulated images (Fig. 6 ▸ *b*). Training was carried out on ten Cori GPU nodes at NERSC, utilizing 80 V100 GPUs, and ran at a speed of 1.6 min per epoch. Multi-node training at NERSC was performed using the *PyTorch* Distributed Data Parallel protocol. Training on a single GPU machine was also tested; using a single V100 GPU, training a model using 43 000 simulated images took 11.5 min per epoch. When training on a single GPU, fewer epochs were required to reach convergence, and the full utility of the Distributed Data Parallel protocol is still being investigated. Table 2 ▸ summarizes the hyperparameters and architectures used for both models.

## 3. Results

### 3.1. Resolution prediction in JUNGFRAU 16M SwissFEL data

**Figure 7:**
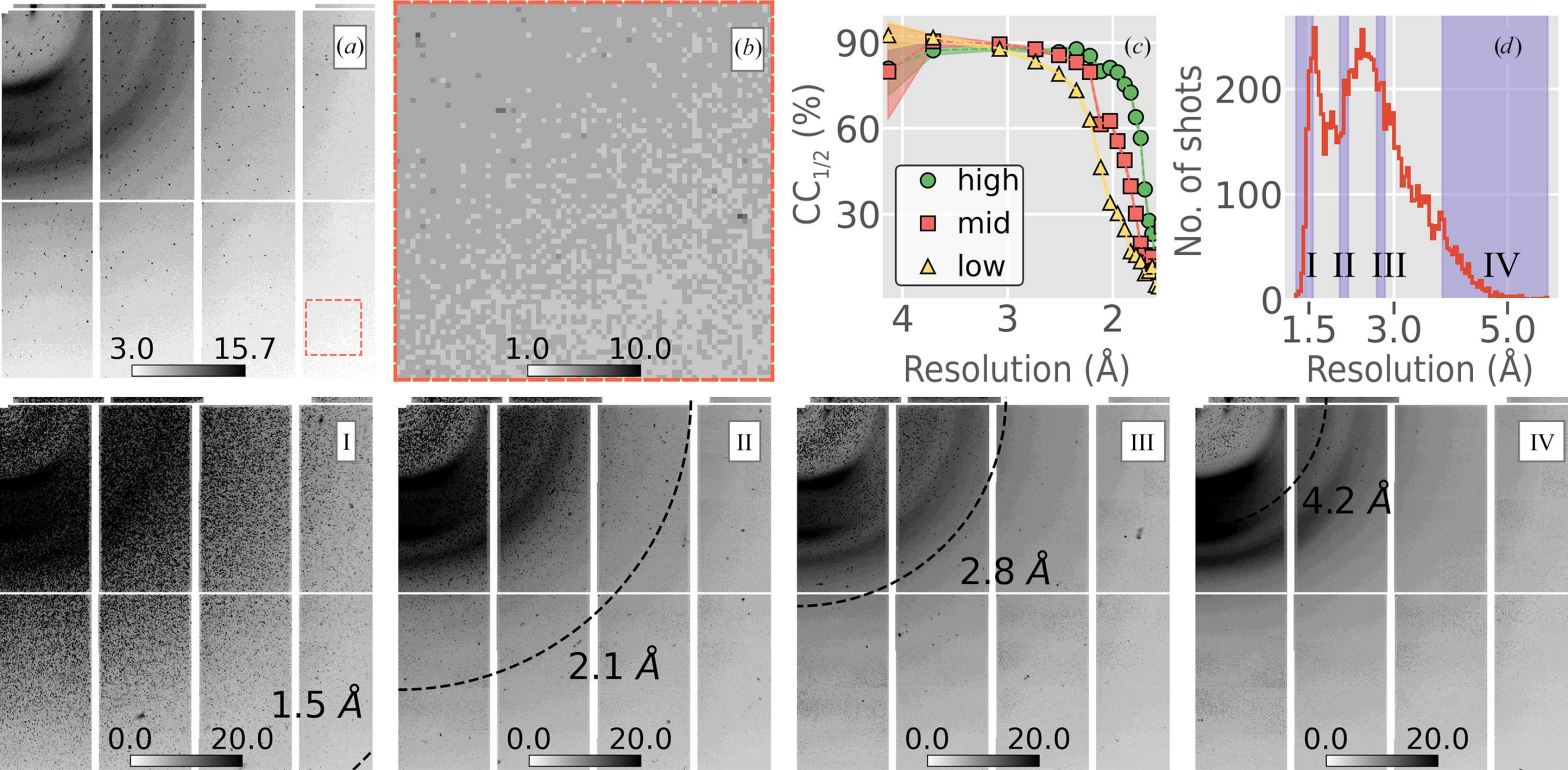
Resolution prediction for JUNGFRAU 16M data collected at the SwissFEL light source. All color bars are in square-root-photon units. (*a*) A quadrant of the JUNGFRAU 16M image (512 × 512 pixels) with the highest predicted resolution (1.3 Å). (*b*) The same as (*a*) but zoomed in to the outer corner of the image, showing high-resolution Bragg peaks. (*c*) CC_1/2_ versus resolution for three different merges. Images were sorted according to *Resonet* predicted resolution and then divided into three groups called ‘high’, ‘mid’ and ‘low’. The high, mid and low groups included images whose *Resonet* predicted resolutions lay in the ranges 1.3–2.5, 2.0–2.9 and 2.5–5.2 Å, respectively. The images in each group were processed with *dials.stills_process* and merged with *cctbx.xfel.merge*, resulting in three CC_1/2_ curves. As shown, images with higher resolutions yielded better CC_1/2_ statistics and indicated that the model can be used to accurately sort images based on resolution. (*d*) Histogram of the resolutions predicted for all 9592 images containing crystals. I–IV show maximum composite images. A maximum composite image is an image whose pixel value is the maximum across a subset of images (for a further description, see Brewster *et al.*, 2019 ▸). In this case, the subsets are those images whose resolutions fell within the shaded regions in (*d*). I, II, III and IV correspond to the intervals 1.3–1.6, 2.0–2.2, 2.7–2.8 and 3.8–5.7 Å and contain 640, 640, 639 and 639 images, respectively. The average resolution in each maximum composite image is labeled by a black dashed circle. The large peaks in these images are from salt crystals or other parasitic scatterers in the beam, and the smaller, more densely packed peaks represent Bragg reflections.

The resolution model was tested on a serial JUNGFRAU 16M data set collected at the SwissFEL light source. CYP121 crystals (Fielding *et al.*, 2017 ▸) were introduced into the SwissFEL SASE (not pink) beam using a tape-drive setup (Fuller *et al.*, 2017 ▸) operated at ambient temperature and pressure. Each JUNGFRAU diffraction image was written to disk as a three-dimensional array (32 × 1024 × 512 pixels); however, our resolution-prediction model expected 512 × 512 quadrant images oriented with the beam center aligned with the first pixel in memory (for example as in Figs. 2 ▸ and 3 ▸). To accommodate the model, each JUNGFRAU image was cast as a two-dimensional array of size 4434 × 4218 and the data were subsequently downsampled into 512 × 512 quadrants (Section 2.2). A resulting JUNGFRAU quadrant is shown in Fig. 7 ▸(*a*). Fig. 7 ▸ describes the results from *Resonet* inferring resolution for the entire data set of 9592 crystal hits. The predicted resolutions were in the range 1.3–5.7 Å (Fig. 7 ▸ *d*) and the resolution obtained from *cctbx.xfel.merge* after processing all 9592 hits was 1.6 Å. It is noteworthy that the resolution model used here was trained on PILATUS 6M and EIGER 16M geometries but was able to estimate accurate resolutions for these JUNGFRAU 16M data without any modifications. *Resonet* overlapping lattice prediction was also tested for these data (see Supplementary Fig. S9). See Supplementary Section S1 for more examples of the application of *Resonet* to XFEL data.

### 3.2. Resolution prediction for SSRL data

**Figure 8:**
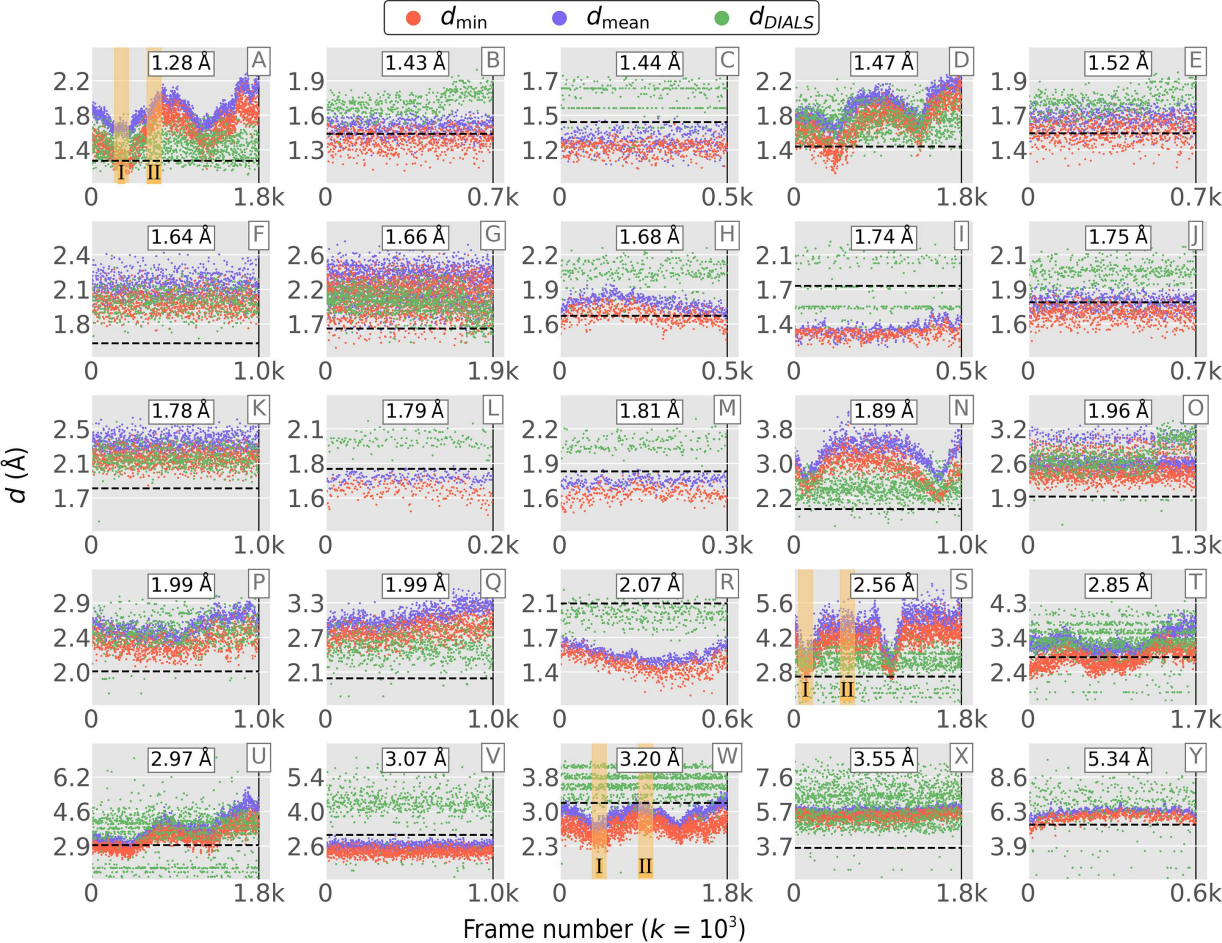
*Resonet* resolution prediction for rotational data sets recorded at the SSRL Structural and Molecular Biology (SMB) beamlines. The horizontal axis is sorted according to collection time. The red and blue markers are the minimum and average inferred resolution per shot (across the four quadrants), respectively, and the green markers are resolution estimates from *DIALS*. Each data set is labeled by a resolution determined using *AIMLESS* (shown at the top of each subplot and indicated by the dashed line) as the point where the overall signal-to-noise ratio of the integrated intensity dipped below 1.5. The yellow-shaded regions labeled I and II in subplots A, S and W correspond to regions where maximum composite images were computed to identify the cause of systematic variation in the *Resonet*-predicted resolution (see Fig. 9 ▸ for the corresponding maximum composite images).

**Figure 9:**
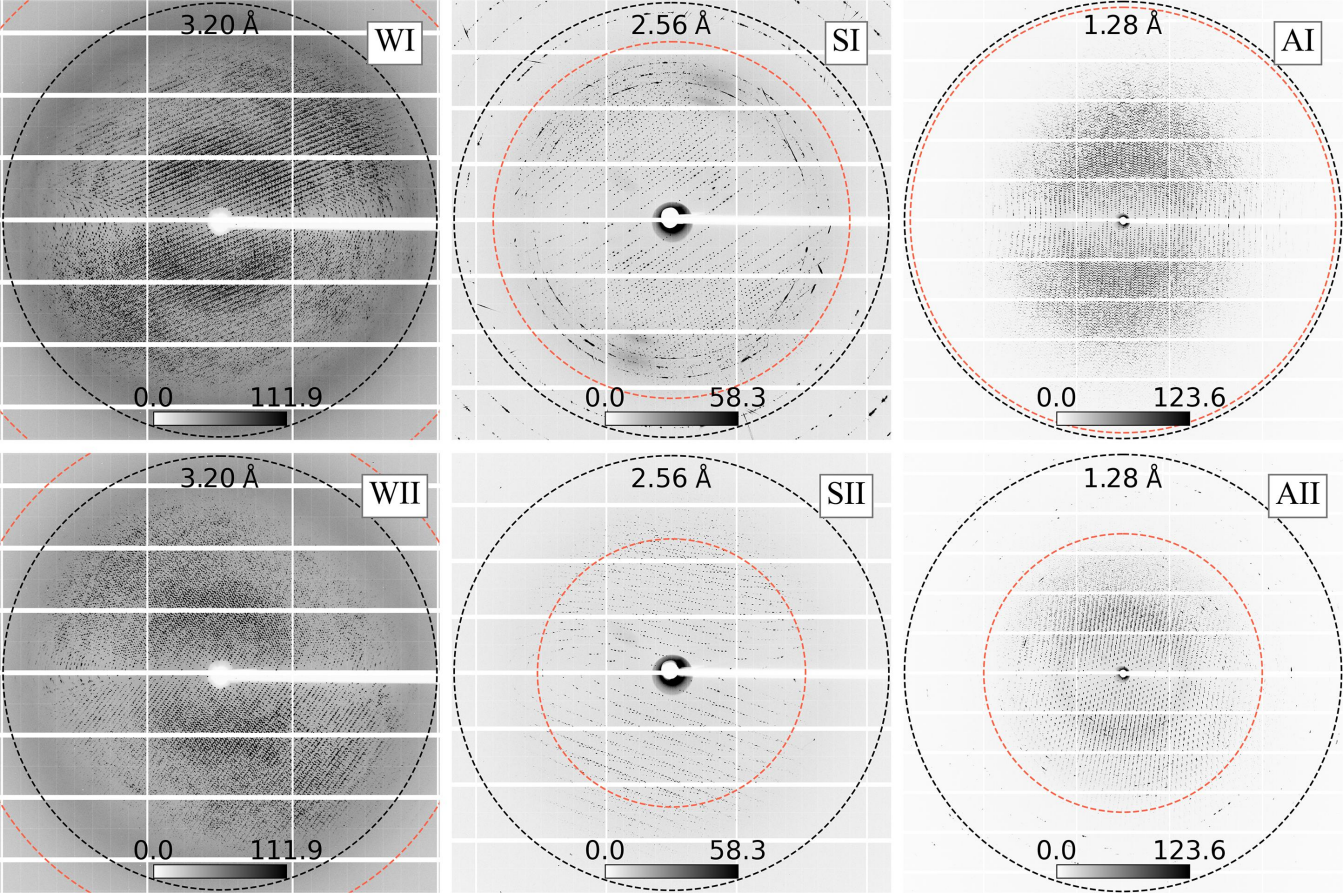
Maximum composite images of sequential groups of SSRL diffraction patterns. The resolution labels 3.20, 2.56 and 1.28 Å correspond to the SSRL data sets that are shown in Fig. 8 ▸ (subplots W, S and A, respectively). The shot ranges over which the maximum composite images were computed are shown in the subplots in Fig. 8 ▸ (W, S and A). Regions labeled ‘I’ correspond to relatively high-resolution estimates and regions labeled ‘II’ correspond to low-resolution estimates from the same data set. The black circles indicate the nominal resolutions of the data sets (taken from the *AIMLESS* logs), and the red circles mark the average resolutions determined by *Resonet* for the images that went into each maximum composite. Different features in the data can influence the *Resonet* resolution. Here, we observe asymmetric diffraction influencing the predicted resolution of the pattern.

*Resonet* resolution inference was performed for 25 rotation data sets obtained at the SSRL SMB beamlines. Table 3 ▸ describes these data sets. Each data set was labeled with an overall resolution, determined from the output of *AIMLESS* (Evans & Murshudov, 2013 ▸) as the point (resolution) where the signal-to-noise ratio of the structure-factor intensity dipped below 1.5. Fig. 8 ▸ shows the *Resonet* resolution versus image number for each of these data sets. For each diffraction image, four resolutions (one per quadrant) were predicted and either the minimum or the mean resolution across the quadrants was taken as the effective resolution (Fig. 8 ▸; red and blue markers, respectively). Also shown in Fig. 8 ▸ is the per-image resolution estimated by *DIALS* (Winter *et al.*, 2018 ▸). In most cases *Resonet* inference worked qualitatively well and trends in *Resonet* resolution inferences were confirmed to be due to changes in diffraction quality and/or anisotropy (Fig. 9 ▸). These synchrotron data represent a large array of experimental conditions, not all of which were captured by our forward model based on *nanoBragg*. The challenge in creating a generalized resolution-prediction model is in preparing the training data and ensuring that they cover the most important scenarios, something that is still under investigation.

**Figure 10:**
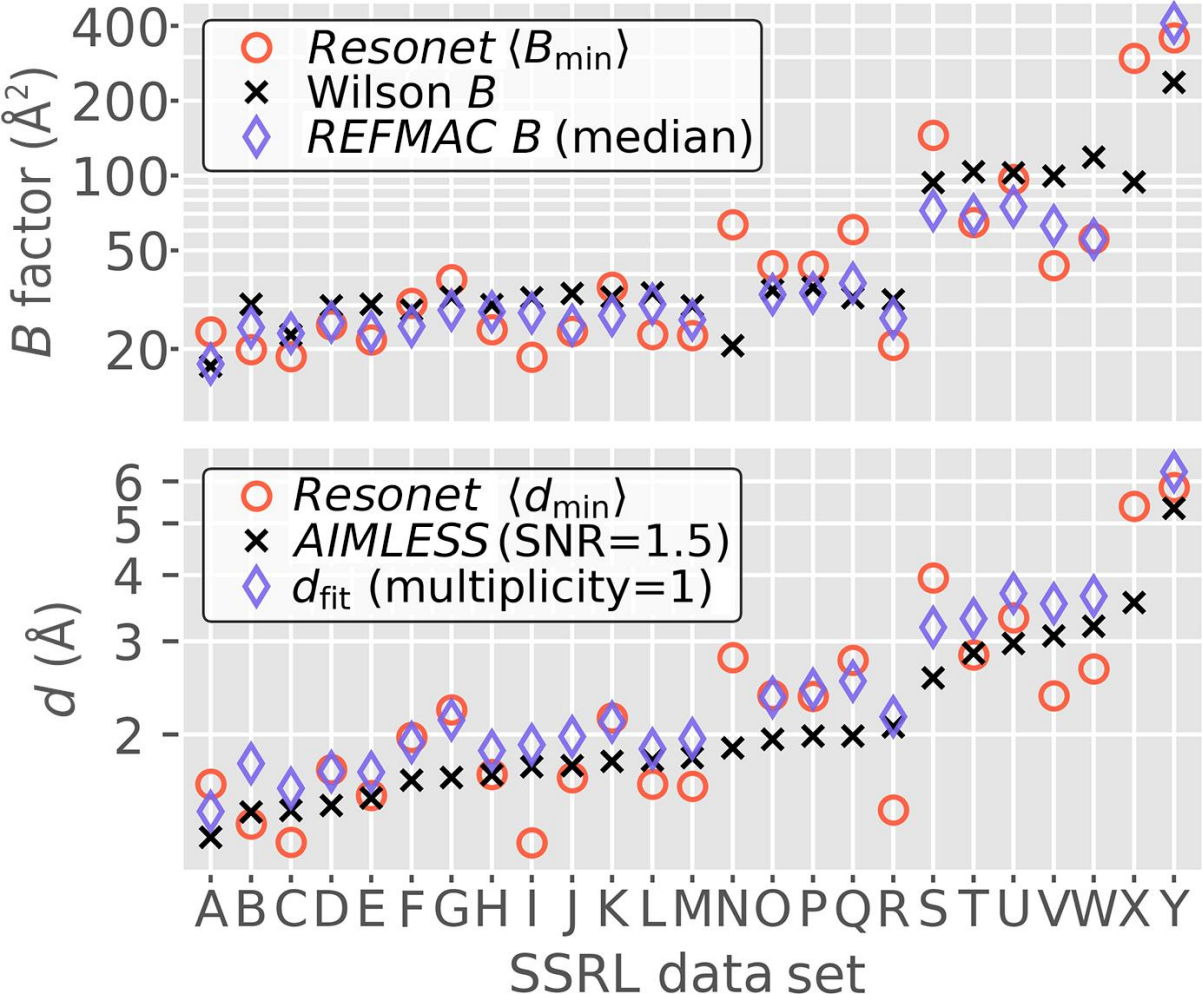
Overall *B*-factor and resolution estimation for each of the SSRL data sets shown in Table 3 ▸ and Fig. 8 ▸. The ‘min’ subscript for the *B* factor (*B*_min_) and resolution (*d*_min_) represents the minimum of the four values inferred across the quadrants of each image and the angle brackets 〈〉 indicate an ‘average over images per data set’ (the number of images in each data set is labeled in Fig. 8 ▸). Top: the overall *Resonet* *B* factor, the Wilson *B* factor (from *AIMLESS*) and the median atomic *B* factor (from structure solution and refinement using *REFMAC*). Bottom: the overall *Resonet* resolution, the resolution determined with *AIMLESS* and a resolution estimate *d*_fit_ based on all processed images but extrapolated to unit multiplicity (for comparison with the *Resonet* inference, which is for a single shot). See Section A2 for a detailed description of *d*_fit_.

As described in Section 2.1.1, the *Resonet* resolution model was designed to infer per-image *B* factors and convert them to resolutions via the relationship shown in Fig. 1 ▸. This relationship is an approximate one (Holton & Frankel, 2010 ▸), hence a comparison between the *Resonet* *B* factors and those derived using other means was warranted (Fig. 10 ▸). For this comparison, the *Resonet* *B* factor of each data set was computed as follows: for each diffraction image in a data set, *Resonet* was used to infer four *B* factors (one per quadrant). *B*_min_ was defined as the minimum *B* factor amongst the quadrants of an image and was then averaged across the data set to obtain the ‘*Resonet B*_min_’ quantity shown in Fig. 10 ▸. We found this correlated best with the Wilson *B* factor (Wilson, 1942 ▸) and the median atomic *B* factor refined using *REFMAC* (Murshudov *et al.*, 2011 ▸).

### 3.3. Overlapping lattice detection in Rayonix 340 data collected at LCLS

To test overlapping lattice prediction, a data set produced using cracked crystals was analyzed with *Resonet*. The data were fixed-target diffraction images collected at 100 K using a goniometer-based setup (Cohen *et al.*, 2014 ▸) at the X-ray Pump Probe (XPP) hutch of LCLS (Chollet *et al.*, 2015 ▸), and the results have previously been published (Artz *et al.*, 2020 ▸). Crystals were translated by 70 µm and rotated between exposures, resulting in 512 diffraction images. Many of the images, however, were collected from volumes of the crystal which featured cracks that gave rise to split and overlapping Bragg peaks. This complicated the analysis originally reported by Artz and coworkers, who visually selected and subsequently processed 122 images which appeared to lack overlapping lattice features.

**Figure 11:**
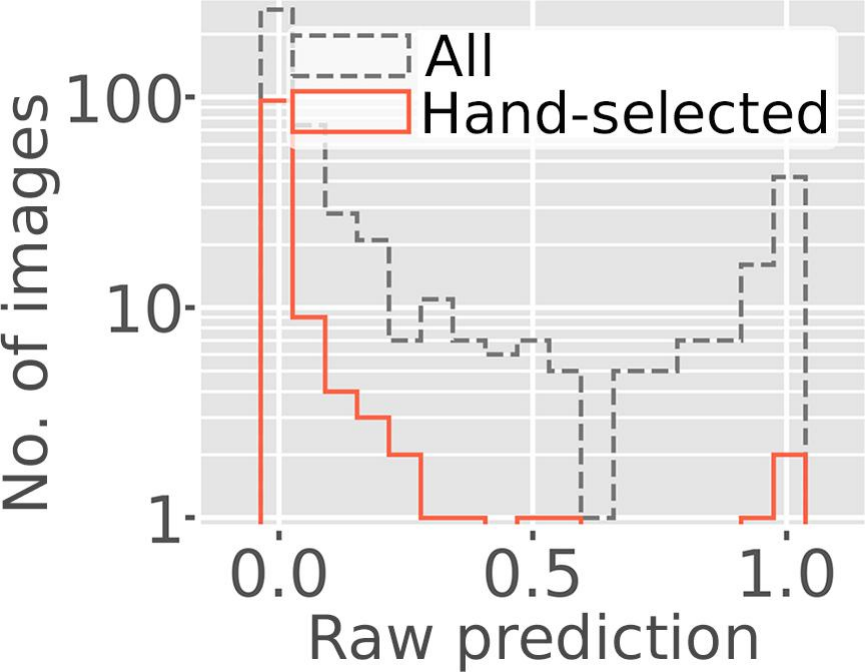
*Resonet* overlapping lattice detection for fixed-target data collected at LCLS (Artz *et al.*, 2020 ▸). The raw prediction corresponds to the probability that the image contains overlapping lattice diffraction; hence, subtracting this number from 1 computes the probability that the image only contains diffraction from a single lattice. The gray histogram represents all 512 images, whereas the red histogram represents the 122 images that were hand-selected for processing in Artz *et al.* (2020 ▸). These hand-selected images were chosen because they resembled good-quality, single-lattice diffraction. Only four of these hand-selected images were predicted by *Resonet* to contain overlapping lattice scattering (with overlapping lattice probability > 50%).

**Figure 12:**
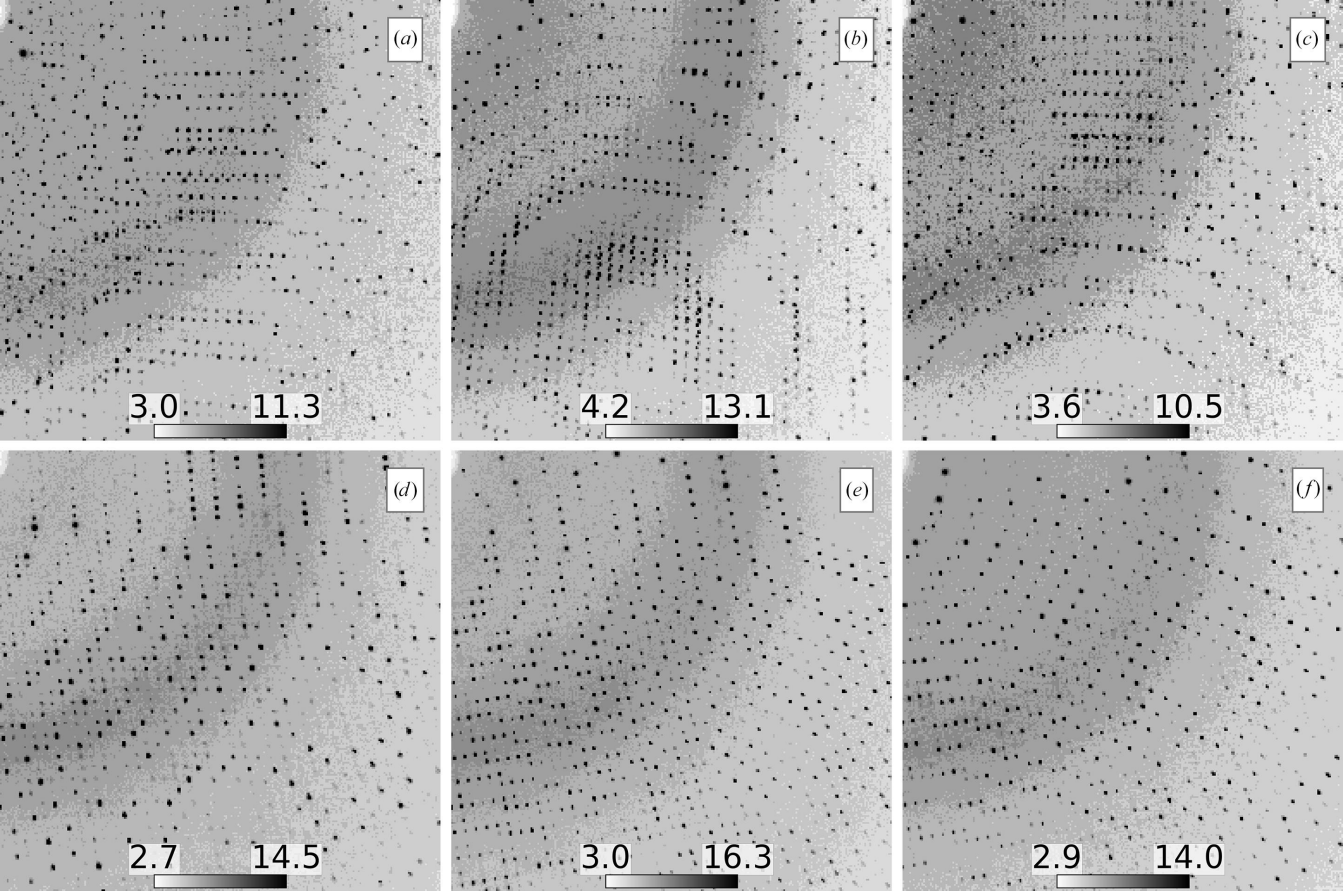
Comparing overlapping-lattice and single-lattice diffraction images from the XPP data set. Images (*a*)–(*c*) were flagged by *Resonet* as containing overlapping lattice diffraction and images (*d*)–(*f*) were flagged as containing diffraction from a single lattice. It is obvious visually that images (*a*)–(*c*) contain more disordered diffraction, indicative of overlapping lattice scattering. Color bars are in square-root-photon units.

**Figure 13:**
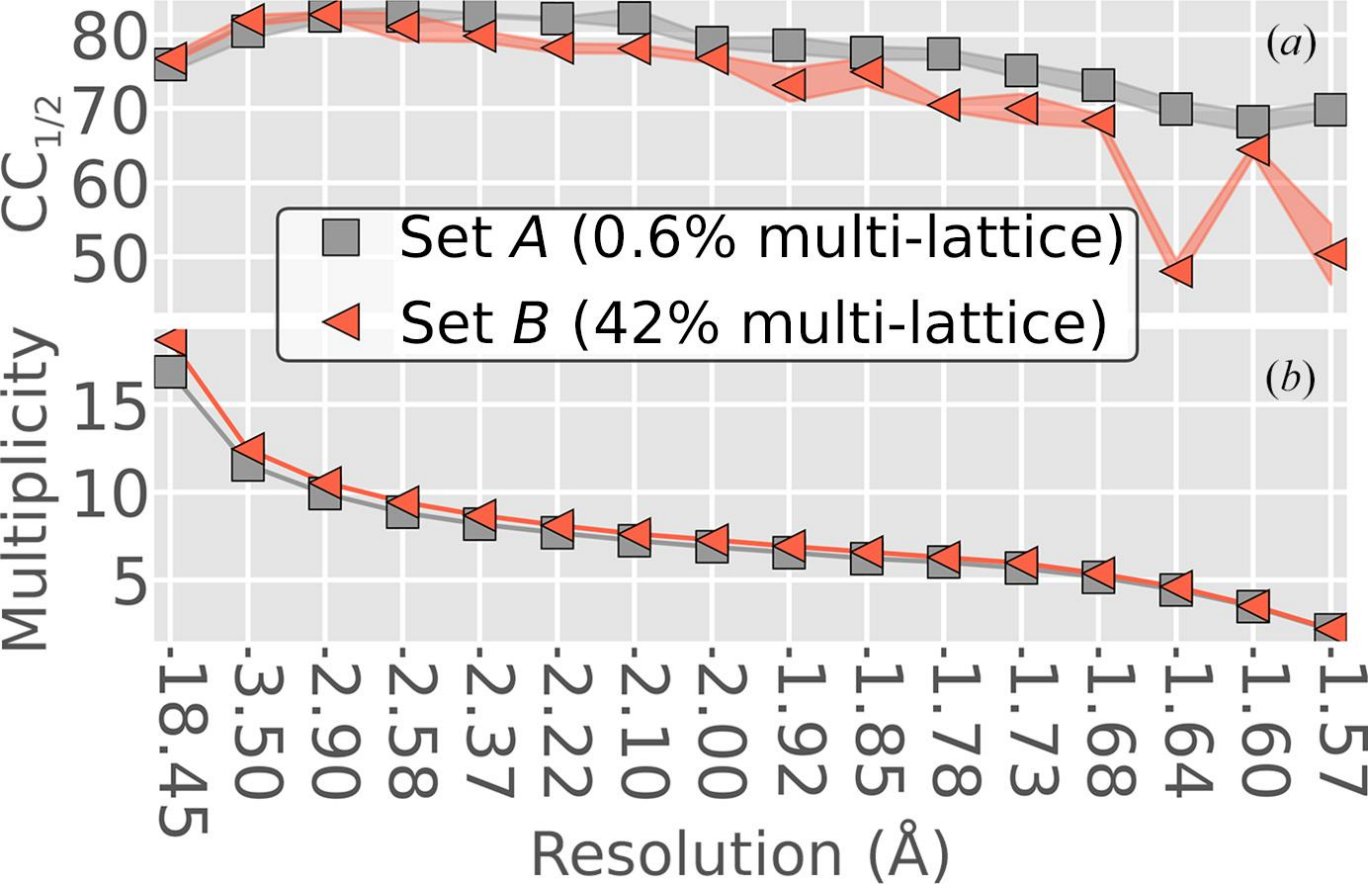
Merging statistics for hydrogenase data collected at XPP. After indexing the images with *dials.stills_process*, they were grouped into two sets according to the probability of each image containing overlapping lattice diffraction (according to *Resonet*). Each set contained 198 images. (*a*) The CC_1/2_ obtained after merging either set using *cctbx.xfel.merge*. CC_1/2_ was computed five times per set with random half-data-set assignments. The markers represent the mean, and the shaded region indicates ±1 standard deviation from that mean. (*b*) The merged multiplicity in the asymmetric unit. Notably, Set *B* had a slightly higher overall multiplicity (7.83 versus 7.36), but a lower CC_1/2_ at high resolution.

For this report, the *Resonet* overlapping lattice model was tested using the entire 512-image data set. The overlapping lattice prediction value, which we call *p_i_* for image *i* (where 0 ≤ *p_i_* ≤ 1), indicated that 420 of the images were single-lattice (*p_i_* < 0.5); this fraction included 118 of the 122 images hand-selected by Artz and coworkers (Fig. 11 ▸). Examples of images flagged as having single or overlapping lattices by *Resonet* are shown in Fig. 12 ▸; in these examples, *Resonet* predictions aligned well with visual inspection. To seek a more quantitative result, the original data were reprocessed with a recently updated version of *dials.stills_process*. Starting with the set of 512 images, 437 were indexed and integrated. Of these images, 41 were removed for having a relatively low number of indexed reflections. The remaining 396 images were sorted according to *p_i_* and split into two data sets of equal size. The 50th percentile of *p_i_* for these data was 0.0265, so we created Set *A* and Set *B*, such that 0 ≤ *p_i_* ≤ 0.0245 for Set *A* and 0.0248 ≤ *p_i_* < 1 for Set *B*. Images in Set *A* and Set *B* had an average overlapping lattice probability *p_i_* of 0.006 and 0.42, respectively. The structure-factor intensities from both sets were merged separately, and the resulting CC_1/2_ statistics are shown in Fig. 13 ▸. Notably, the CC_1/2_ was lower at wider scattering angles for the set that included more overlapping lattice diffraction (Set *B*). This is in line with the general assumption that split and/or superimposed Bragg spots from overlapping lattices are problematic for most data-processing software. We emphasize that the detection of overlapping lattices within diffraction data using conventional tools typically requires indexing, for example, to calculate the fraction of observed Bragg peaks that are indexable (see Supplementary Figs. S3–S7). In contrast, *Resonet* only uses the raw pixel values. For completeness, *Resonet* resolution estimation for these data is shown in Supplementary Fig. S10. For additional examples of the application of *Resonet* overlapping estimation to XFEL data, see Supplementary Sections S1.2 and S1.3 and Supplementary Figs. S3–S6.

### 3.4. Overlapping lattice detection in diffraction from SSRL

**Figure 14:**
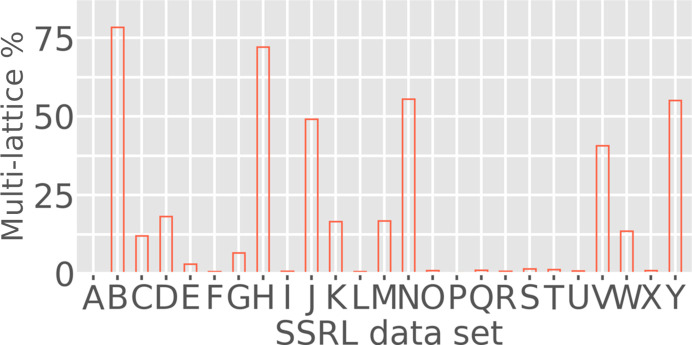
*Resonet* overlapping lattice detection in the 25 SSRL data sets described in Table 3 ▸ and shown in Fig. 8 ▸. The *y* axis here indicates the probability that an image from the data set contains overlapping lattice diffraction.

**Figure 15:**
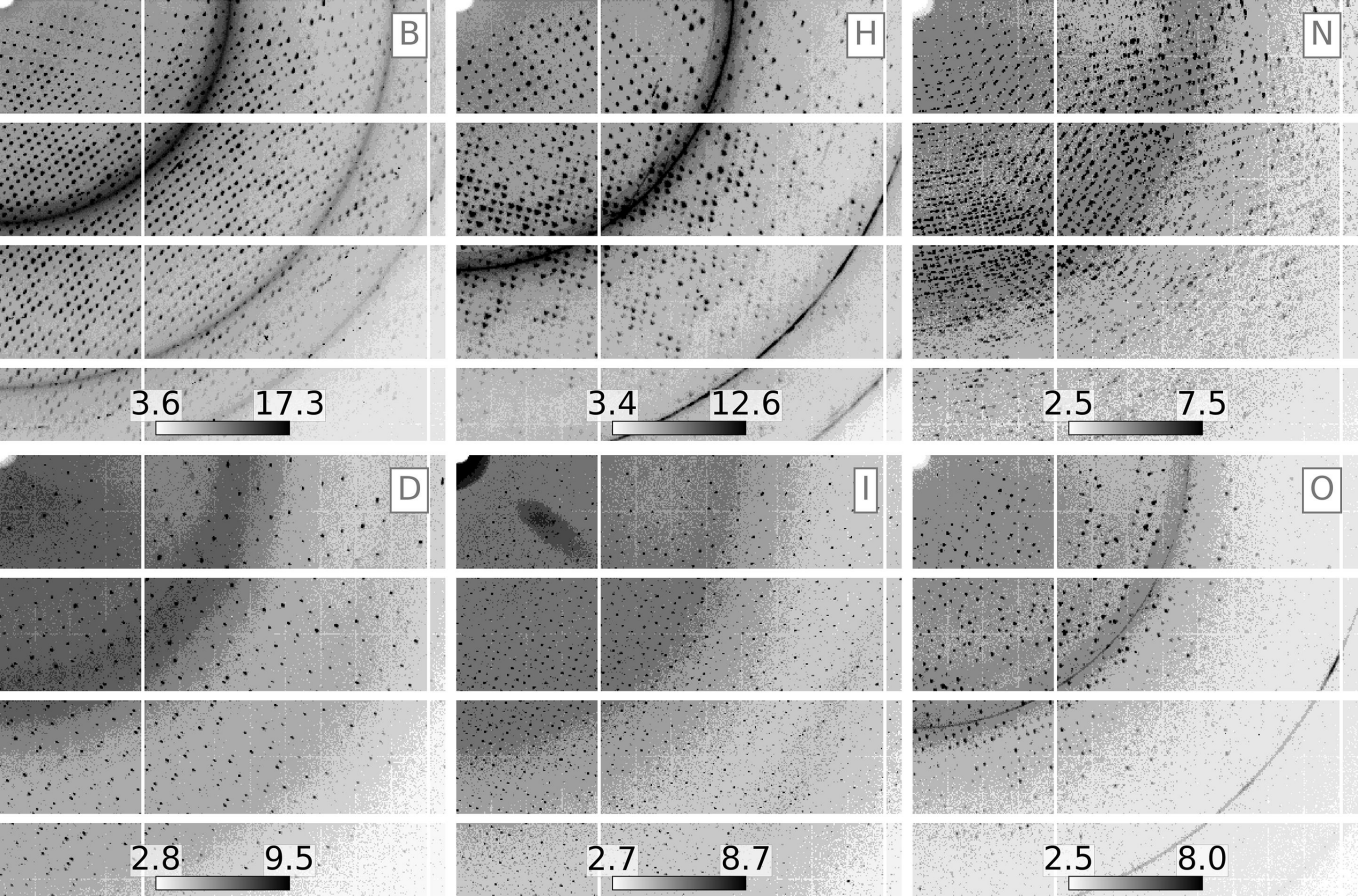
Maximum composite images for the first 20 exposures (4° total rotation) from the SSRL data sets indicated by the subplot labels B, H, N, D, I and O. Based on *Resonet* overlapping lattice detection (see Fig. 14 ▸) it was known that data sets B, H and N had a high chance of containing overlapping lattice scattering (80%, 74% and 57%, respectively). On the contrary, data sets D, I and O had a low chance of containing overlapping lattice scattering (12%, 0% and 0%, respectively), but were similar in setup to experiments B, H and N. All color bars are in ‘square-root-photon’ units. While data set N clearly exhibited diffraction indicative of overlapping lattice features from separate protein crystals, it appeared that data sets B and H contain more subtle features (streaks and Bragg peaks from ice and/or salt) that cause *Resonet* to infer the presence of overlapping lattices.

*Resonet* overlapping lattice detection was performed on the data sets outlined in Table 3 ▸. The results are summarized in Fig. 14 ▸. From these results, it was concluded that four of the data sets (B, H, N and Y) had a majority amount of overlapping lattice scattering (>50% of the images). A closer look at images from these data sets revealed features indicative of overlapping lattice scattering, as shown in Fig. 15 ▸. Notably, the overlapping lattice-detection model was trained on simulated images in the Rayonix 340 detector format used during the XPP data collection discussed above; however, it worked well on these SSRL data sets consisting of PILATUS 6M and EIGER 16M images. This seems to indicate that the overlapping lattice features that the model looks for are related to the Bragg peak profiles, and are mostly independent of the underlying detector geometry. One complication appears to be overlapping lattice features appearing in ice and salt diffraction. Although these ice and salt features can be masked, future versions of *Resonet* will infer their presence, characteristics and severity, and attempt to decouple them from overlapping lattice inference.

### 3.5. Implementation at SSRL beamlines

**Figure 16:**
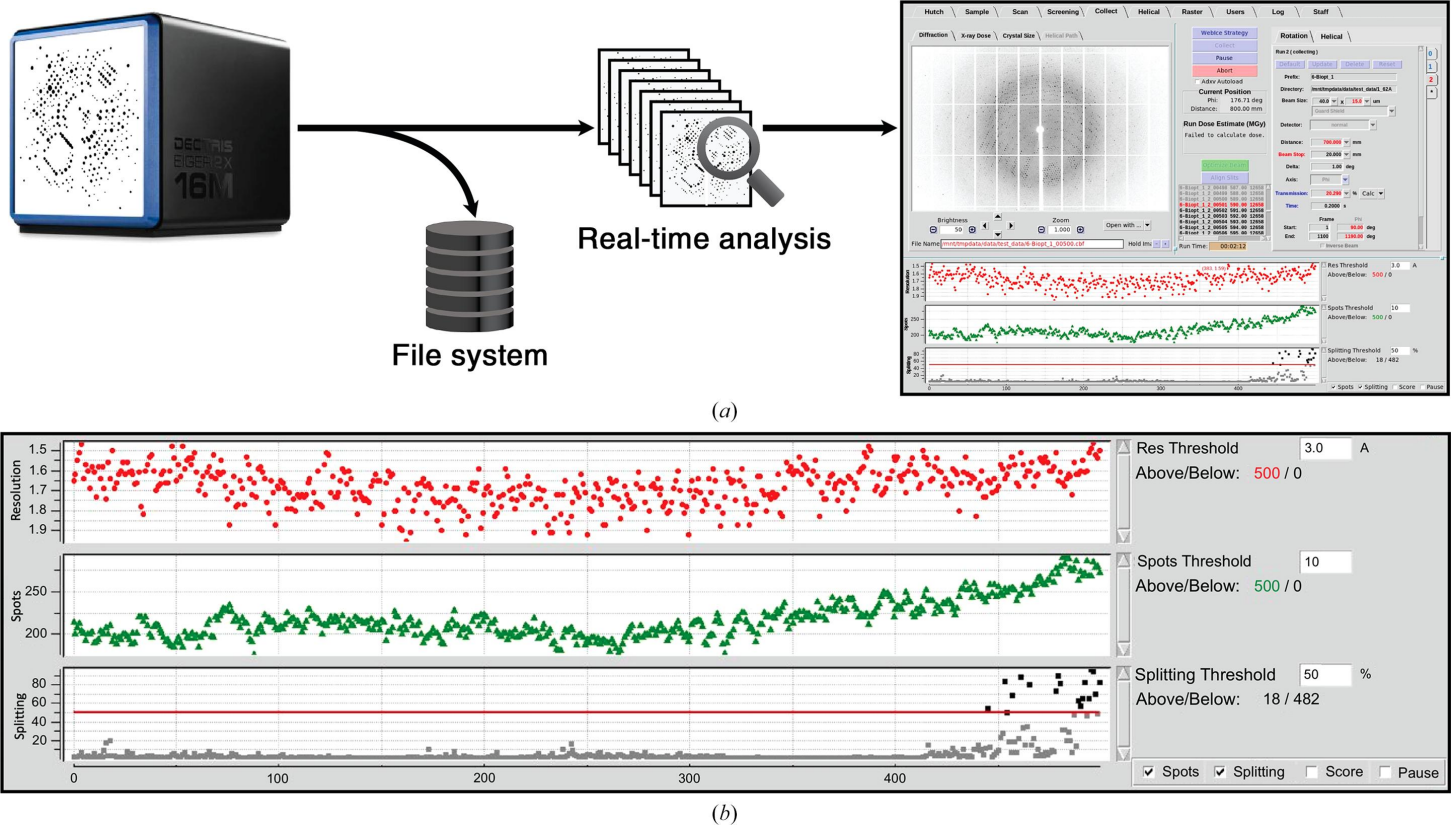
Live X-ray diffraction-image analysis with *Interceptor*. (*a*) During data collection, the images are recorded on the file system and subsequently forwarded to a set of parallel processing modules. The results are then forwarded to the *Blu-Ice* beamline-control software and user interface. (*b*) Users can visualize the results in a configurable strip chart, which is updated as the data are collected. The configuration shown consists of a plot of resolution (top, red), peak count (middle, green) and overlapping lattice probability (called ‘splitting’; bottom, black/gray) versus image number. Near the end of the run, the ‘splitting’ trace indicated the presence of overlapping crystal diffraction. Also at this point, the number of spots (green) increased, but the resolution remained unchanged, as expected.

Both the resolution and overlapping lattice-prediction models are currently implemented in the live X-ray diffraction analysis program *Interceptor* and the SSRL beamline-control software *Blu-Ice* (McPhillips *et al.*, 2002 ▸). *Interceptor* evaluates all images collected at SSRL macromolecular crystallography beamlines and sends the results to *Blu-Ice*, which updates a chart of relevant metrics in real time for users to see (Fig. 16 ▸). As *Interceptor* was written in Python, a basic interface to embed *Resonet* into Python applications was created (see https://github.com/ssrl-px/resonet), and this same interface should also be usable by other monitoring software, for example *OM* (Mariani *et al.*, 2016 ▸). Further details of *Interceptor* are discussed in Section A3.

### 3.6. Processing times on a GPU

The *Resonet* resolution model was carefully timed using a 24-core (Intel Xeon Gold 6126 2.6 GHz) machine running CentOS 7 with an Nvidia A100 GPU. The GPU was utilized by multiple cores in parallel, and parallelization was performed by evenly dividing the diffraction images over cores using the message-passing interface (MPI) protocol. The results are shown in Table 4 ▸; using a single GPU shared amongst multiple processes greatly improved the inference time and overall throughput. With this one machine, using all 24 cores, the A100 and only using one quadrant for inference, EIGER 16M images were processed at 97.7 Hz and PILATUS 6M images at 261 Hz, including the time taken to read the images from disk using the FabIO library (Knudsen *et al.*, 2013 ▸). It is expected that these times will vary depending on the way that raw pixels are handled in disk and RAM, and whether the detectors must first write to disk before moving data to processing machines. Without the GPU, these processing rates decreased to 18.1 Hz (EIGER 16M) and 20.4 Hz (PILATUS 6M). These results, however, suggest that GPU machines have great potential for providing faster real-time feedback to users. Additional timing tests are shown in Supplementary Fig. S8.

### 3.7. Quadrant variation

**Figure 17:**
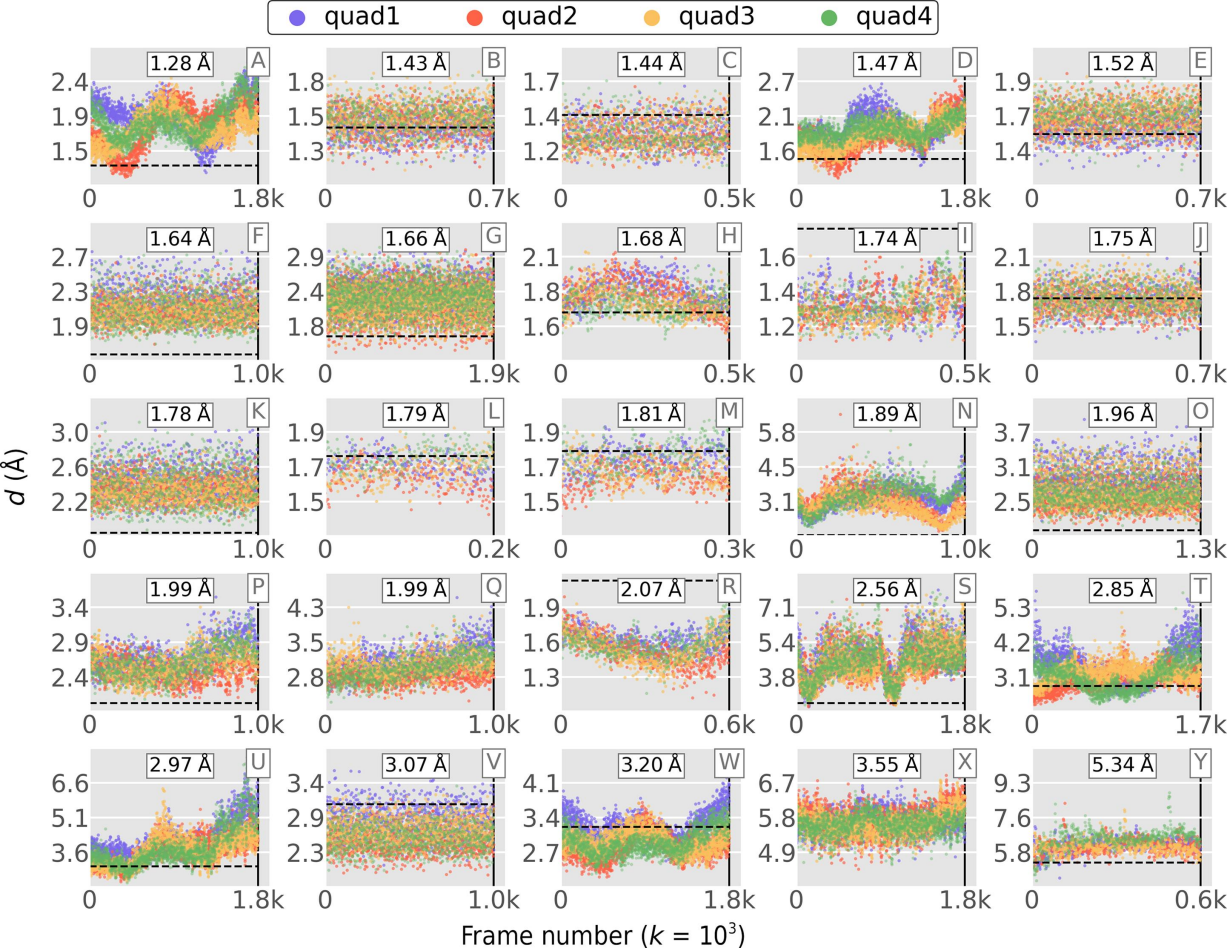
As Fig. 8 ▸, but separate inferences are shown for each quad.

Due to the timing-test results shown in Table 4 ▸, for high-frame-rate experiments it may sometimes be beneficial to use a single quadrant for inference. Indeed, a single quadrant was used for all of the overlapping lattice results shown in this report. However, for the resolution inference results shown in Figs. 8–10, all four image quadrants were used to infer resolution separately, and the mean (or minimum) was then taken as the effective resolution. Looking at the entire image to gauge its resolution is perhaps the most accurate approach, but it is instructive to explore the variation in resolution across quadrants. This is shown in Fig. 17 ▸ for the 25 SSRL data sets from Table 3 ▸ (and Fig. 8 ▸). In most of the data sets the resolutions were similar regardless of quadrant; however, anisotropic diffraction and inaccurate beam centering can both influence resolution inference in individual quadrants (see, for example, Fig. 17 ▸ *a*). Future versions of *Resonet* models will be trained on more diverse data sets to yield even more precise resolution estimates. It is intriguing to postulate that these models could be trained to recognize diffraction anisotropy and incident beam misalignment from a single quadrant.

## 4. Discussion

AI as a tool is inherently tied to automation. The central utility of computers is to enhance the human experience by automating routine tasks, and this goes for crystallographers as well. Indeed, data analysis at SR crystallography beamlines has increasingly become automated (Cornaciu *et al.*, 2021 ▸; Douangamath *et al.*, 2021 ▸; Tsai *et al.*, 2013 ▸), and this is also true for XFEL experiments. For example, during two recent LCLS experiments targeting small molecules and viral COVID-19 proteins (Blaschke *et al.*, 2021 ▸), data were recorded at SLAC and automatically transferred using the *XROOT* protocol to the National Energy Research Scientific Computing Center (NERSC) for high-performance computing. Data-processing jobs were submitted to NERSC compute nodes remotely by the *cctbx.xfel* application (Brewster *et al.*, 2019 ▸), and preliminary structure solutions were automatically uploaded to a web server for experimenter assessment in as little as 10 min after 120 Hz data collection began. This did require initial user inputs for indexing, integration, merging and structure refinement, but with the addition of new AI programs (Ke *et al.*, 2018 ▸; Rahmani *et al.*, 2023 ▸) to screen for diffraction, and our present body of work that uses AI to characterize diffraction, we are edging away from requiring user inter­actions for serial data processing.

One drawback of using these supervised learning approaches is the sensitivity to training-data content. Indeed, in Ke *et al.* (2018 ▸) and Rahmani *et al.* (2023 ▸) the authors concluded that their training data sets did not readily adapt to new data collected under different experimental conditions or using different setups. We have seen training-set bias in our own work as well. The benefit of our simulation-to-model approach is that the simulations are fully within our control, allowing us to readily expand training data sets to adjust for shortcomings and to adapt to new experimental parameters and setups. As an example, we applied the models trained here to serial crystallography data from the early-generation CSPAD camera (Hart *et al.*, 2012 ▸) used in Boutet *et al.* (2012 ▸) and found that they performed poorly (Supplementary Section S1.2). We suspected that this was because the data collection by Boutet and coworkers, performed at the LCLS, used a vastly different experimental geometry (9.4 keV photons, 93 mm sample-to-detector distance). By simply retraining the resolution-prediction model on synthetic data simulated in this regime, we were able to accurately estimate the resolution for these images (Supplementary Figs. S1–S3). In our experience, *Resonet* was sensitive to the data that it was trained on in complex, obscure ways. By increasing the training-data diversity, we could seek to train a single model to work in all conceivable diffraction scenarios, but it would perhaps be more computationally efficient to train smaller models targeting specific scattering geometries that can be used as needed. Further, with the *Resonet* framework, we are well positioned to begin exploring the prediction of other interesting experimental parameters. We are actively exploring the use of *Resonet* models to determine the incident beam position on the detector, and the preliminary results are encouraging. These models could then be used to warn users when the detector or beam geometry is misaligned. In addition to providing real-time feedback, we expect that *Resonet* will reduce the time and effort required to aptly process challenging data sets. *Resonet* can accurately detect and flag problematic diffraction images, such as highly anisotropic images or those containing parasitic amounts of overlapping lattice diffraction. The inclusion of problematic diffraction images reduces the quality of merged data, making structure determination (especially by *ab initio* phasing) difficult or impossible. We expect that *Resonet* will be key to identifying which diffraction images should be included and processed to yield optimal merged data sets.

AI models are already being used to scale and merge structure-factor intensity measurements (Dalton *et al.*, 2022 ▸). AI tools such as *Resonet* can potentially be used to inform users of progress towards full data sets or how to adjust the beamline parameters to optimize the chances of experimental success. The presented work demonstrates two ways in which AI models might aid crystallographers, but additional models (for example auto-indexers) can and should be developed. Future work to utilize AI for diffraction processing will lead to better results and higher throughput experiments at crystallography beamlines in general.

## 5. Availability of *Resonet*

Installation instructions, and tutorials for simulating images, training a *Resonet* model and applying existing *Resonet* models (including those used for this report), are available at https://github.com/ssrl-px/resonet.

## 6. Related literature

The following references are cited in the supporting information for this article: Maia (2012 ▸), Sellberg *et al.* (2014 ▸) and Nam & Cho (2021 ▸).

## Supplementary Material

Processing serial crystallography data from CXIDB; Supplementary Figures. DOI: 10.1107/S2059798323010586/qi5003sup1.pdf

## Figures and Tables

**Table 1:** Simulation properties corresponding to Fig. 2 ▸

Label in Fig. 2 ▸	Resolution (Å)	Distance (mm)	Background scale
A	2.06	217	1.25
B	1.71	221	1.25
C	2.46	240	1.25
D	12.8	253	1
E	5	224	0.05
F	1.43	253	1
G	1.63	298	0.01
H	2.5	250	1
I	1.9	225	1
J	6.82	274	1.25
K	1.9	293	1.25
L	2.4	260	0.02
M	19.2	238	1
N	2.84	264	0.1
O	2.07	222	1

**Table 2:** Model-fitting details SGD, sparse-gradient descent; MAE, mean absolute error; BCE, binary cross entropy.

Predictor type	Resolution	Overlapping lattice
ResNet parameters	25550760	21791400
Total parameters	25650961	21891601
Optimizer	SGD	SGD
FC1 dropout	No	Yes
Training images	292500	117000
Momentum	0.9	0.9983
Weight decay	N/A	2.5 × 10^−4^
Loss function	MAE	BCE
Learning rate	6 × 10^−3^	1.04 × 10^−3^

**Table 3:** SSRL crystallography data sets tested with *Resonet*

Data set	*AIMLESS *resolution (Å)	Distance (mm)	Detector	Unit-cell volume (Å^3^)	Space group
A	1.28	200	PILATUS 6M	1.89 × 10^−5^	*P*1
B	1.44	250	PILATUS 6M	1.45 × 10^−6^	*I*4_1_22
C	1.43	250	PILATUS 6M	1.51 × 10^−6^	*I*4_1_22
D	1.47	250	PILATUS 6M	2.66 × 10^−5^	*C*121
E	1.52	250	PILATUS 6M	1.45 × 10^−6^	*I*4_1_22
F	1.64	300	PILATUS 6M	1.47 × 10^−6^	*I*4_1_22
G	1.68	300	PILATUS 6M	1.48 × 10^−6^	*I*4_1_22
H	1.74	300	PILATUS 6M	1.44 × 10^−6^	*I*4_1_22
I	1.66	300	PILATUS 6M	1.51 × 10^−6^	*I*4_1_22
J	1.79	300	PILATUS 6M	1.44 × 10^−6^	*I*4_1_22
K	1.81	300	PILATUS 6M	1.51 × 10^−6^	*I*4_1_22
L	1.78	300	PILATUS 6M	1.44 × 10^−6^	*I*422
M	1.75	250	PILATUS 6M	1.47 × 10^−6^	*I*4_1_22
N	2.07	300	PILATUS 6M	2.41 × 10^−5^	*P*12_1_1
O	1.96	300	PILATUS 6M	1.50 × 10^−6^	*I*4_1_22
P	1.89	275	EIGER 16M	1.51 × 10^−6^	*I*4_1_22
Q	1.99	300	PILATUS 6M	1.46 × 10^−6^	*I*4_1_22
R	1.99	300	PILATUS 6M	1.47 × 10^−6^	*I*222
S	2.56	350	PILATUS 6M	2.27 × 10^−5^	*P*4_1_2_1_2
T	2.85	300	PILATUS 6M	9.11 × 10^−5^	*P*2_1_2_1_2_1_
U	3.07	300	PILATUS 6M	1.06 × 10^−6^	*P*2_1_2_1_2_1_
V	2.97	350	PILATUS 6M	1.52 × 10^−6^	*I*222
W	3.20	400	PILATUS 6M	5.18 × 10^−6^	*P*2_1_2_1_2_1_
X	3.55	350	EIGER 16M	2.83 × 10^−6^	*P*4_1_2_1_2
Y	5.39	400	PILATUS 6M	1.48 × 10^−6^	*I*4_1_22

**Table 4:** Downsample and inference time tests on a single Nvidia A100 GPU All times are in milliseconds, normalized by the number of MPI ranks. They therefore represent the effective per-image throughput. Downsample times are medians over SSRL data sets P (EIGER 16M) and D (PILATUS 6M). Standard deviations are shown in parentheses. CPU-only times are shown for reference in the two rightmost columns.

No. of MPI ranks†	No. of quadrants	Detector	Downsample GPU	Inference GPU	Downsample CPU	Inference CPU
8	1	EIGER 16M	2.8 (0.1)	1.0 (0.2)	3.8 (0.5)	70.9 (3.8)
8	4	EIGER 16M	8.8 (0.5)	1.2 (0.4)	16.5 (2.3)	287.6 (21.5)
24	1	EIGER 16M	1.8 (0.2)	0.5 (0.2)	2.5 (0.3)	42.1 (2.6)
8	1	PILATUS 6M	0.9 (0.1)	0.9 (0.1)	1.5 (0.1)	69.9 (3.4)
8	4	PILATUS 6M	2.3 (0.2)	1.2 (0.5)	6.1 (0.6)	290.4 (28.8)
24	1	PILATUS 6M	0.5 (0.1)	0.9 (0.4)	0.9 (0.1)	45.2 (2.3)

† This is equivalent to the number of processors working in parallel. For the GPU tests, these processes equally shared the A100.

## Appendix A Simulation details

### A1.1. Crystal models

For each simulated image, a crystal and list of structure-factor intensities were modeled using a randomly chosen PDB entry from the following list: 1h74, 1hk5, 1keq, 1ktc, 1lbv, 1nne, 1pdv, 1qtx, 1r03, 1rlk, 1sg8, 1uic, 1uv7, 1vh6, 1xrt, 1yj1, 1yo6, 1z35, 1z6s, 2ar6, 2bh4, 2cc3, 2hu3, 2hyf, 2i8d, 2ibm, 2itu, 2nrz, 2pkg, 2qa4, 2qex, 2qma, 2qt4, 2vj3, 2vuy, 2wox, 2wyf, 2x8i, 2xh6, 2y8k, 2ycf, 2zg2, 2znt, 2zry, 3agy, 3ch7, 3cma, 3cpw, 3dll, 3dxj, 3e6l, 3fj8, 3fl2, 3fyx, 3g8y, 3hfp, 3hxf, 3ilo, 3int, 3k6n, 3l89, 3lke, 3lz7, 3n0w, 3nxs, 3oj1, 3t4x, 3tuu, 3u7s, 3uh4, 3uhr, 3vgd, 3woz, 3wpz, 3zbs, 3zg2, 4arq, 4cbc, 4ctn, 4dvn, 4e6i, 4f3x, 4fhm, 4gyk, 4j20, 4m5i, 4m97, 4o09, 4o7s, 4p9h, 4pgu, 4px8, 4qxq, 4rmx, 4wd2, 4xbe, 4xxo, 4ypu, 4z40, 5al4, 5aoo, 5avi, 5dt6, 5g4e, 5g52, 5j77, 5jit, 5o99, 5p9i, 5pjt, 5v5k, 5v5v, 5vn7, 5vn9, 5wqg, 5xg2 and 6csc. These PDB files covered the set of space groups *P*3, *P*3_1_12, *R*3:*H*, *P*6, *P*4_1_2_1_2, *P*3_2_21, *P*321, *P*42_1_2, *C*121, *P*41, *P*4_2_2_1_2, *P*3_1_21, *C*222_1_, *R*32:*H*, *P*4_1_32, *P*2_1_2_1_2_1_, *P*6_1_22, *I*222, *P*4_3_2_1_2, *P*6_1_, *P*1, *P*6_5_22, *I*2_1_2_1_2_1_, *P*3_2_, *P*2_1_2_1_2, *P*12_1_1, *F*432, *P*2_1_3 and *I*23 and had unit cells ranging in volume from 49 800 to 48 400 000 Å^3^. The crystal size was set to 25 µm and the average mosaic domain size was randomly set to 0.05, 0.1 or 0.15 µm. For the resolution training data the angular mosaic spread of each crystal was randomly chosen in the interval 0.05–1°, and for the overlapping lattice training data this range was 0.001–0.01°.

### A1.2. Detector models

For resolution training data sets, each diffraction pattern was simulated onto either an EIGER 16M or a PILATUS 6M detector format and the detector distance was randomly sampled in the interval 200–300 mm. For the overlapping lattice-detection training data, a Rayonix 340 format was used and the detector distance was fixed at 240 mm (according to the experimental geometry that it was modeled after). It was originally planned to retrain an overlapping lattice predictor using other detector models (for example EIGER and PILATUS), but the model trained on Rayonix data performed sufficiently in practice when applied to images from other detector models. In each simulated detector image, a randomly sized circle of pixels (<15 mm) was masked to simulate a beam stop. Also, for each image a random selection of 0–5 pixels was chosen and the pixel values were set to 2^16^ photons to simulate ‘hot’ pixels. A second random selection of up to 124 pixels was chosen and the pixel values were set to 0 to simulate ‘bad’ pixels.

### A1.3. Beam model

The total photons per simulated image was 4 × 10^11^ and each photon had a wavelength of 0.9795 Å. The incident beam had a spot size of 30 µm and a divergence of 0.02 mrad.

### A1.4. Background scattering

For each simulated image, we computed scattering from 5 mm of air, 25 µm of water and 25 µm of a randomly chosen parasitic source (for example glycerol, sucrose, PEG, MDP, DPM, paper, tape or ice). These background components were summed and then added to the Bragg scattering, but with a randomly chosen scale factor (between 0.0125 and 1.25) to simulate different experimental conditions and background levels.

### A1.5. Simulation timing

Forward-scattering simulations were carried out at NERSC using the Perlmutter cluster. Two batch jobs were used to simulate the resolution training data (one per detector model). Each job utilized 64 Perlmutter GPU nodes, each with four A100 GPUs, and 16 physical cores per node (four cores per GPU). In this configuration, the 200 000 PILATUS 6M images and 125 000 EIGER 16M images were simulated in approximately 60 and 90 min, respectively.

### A2. Computing *d*_fit_

In order to extrapolate the resolution *d* of a merged data set to that of a single image *d*_fit_, the resolution-dependence of the signal *I*, noise σ_*I*_ and multiplicity *m* of unmerged spot intensities must be taken into account. Here, the value for *m* was taken from the outer resolution bin of the merged data. For *I* and σ_*I*_ individual, Lp-corrected spot-intensity data from *XDS_ASCII.HKL* were fitted to straight lines on a plot of *d* ^−2^ versus ln(*I*) or ln(σ_*I*_), which is analogous to a Wilson plot. The point where the two lines crossed at *Im* ^1/2^ = 1.5σ_*I*_ was found to be in excellent agreement with the resolution *d* reported by *AIMLESS* using the signal/noise = 1.5 criterion, and the point where *I* = 1.5σ_*I*_ was taken as *d*_fit_, the resolution at unit multiplicity.

### A3. Details of the *Interceptor* data monitor for the SSRL SMB beamlines

*Interceptor* is a live data-collection monitoring program that is implemented on all SSRL macromolecular crystallography beamlines. It was designed to (i) balance the load among many distributed image-analysis workers, (ii) minimize disk I/O, (iii) handle scenarios of worker shortage at peak capacity and (iv) provide the workers with immediate access to individual images before they are incorporated into an aggregate file format, for example HDF5. The architecture was implemented using the ZeroMQ messaging library, with available workers requesting images using the ZeroMQ REQ protocol and the data-collection software forwarding the data via the REP protocol while images are written to disk. The original version of *Interceptor* relied on algorithms implemented in *DIALS* (Winter *et al.*, 2018 ▸); recently, we have begun replacing these algorithms with *Resonet* and testing this configuration during live X-ray crystallography experiments.
