# Supplementary material for: Deep residual networks for crystallography trained on synthetic data
Source: Acta Crystallogr D Struct Biol. 2024 Jan 1;80(Pt 1):26–43. doi: 10.1107/S2059798323010586 (PMC10833344; doi:10.1107/S2059798323010586)
Supplement: Supplementary file 1 [file d-80-00026-sup1.pdf]

Acta Cryst D STRUCTURAL BIOLOGY

Volume 80 (2024)

Supporting information for article:

Deep residual networks for crystallography trained on synthetic data

Derek Mendez, James M. Holton, Artem Y. Lyubimov, Sabine Hollatz, Irimpan I. Mathews, Aleksander Cichosz, Vardan Martirosyan, Teo Zeng, Ryan Stofer, Ruobin Liu, Jinhu Song, Scott McPhillips, Mike Soltis and Aina E. Cohen

Contents

S1 Processing serial crystallography data from CXIDB **2**

S1.1 Retraining the *Resonet* resolution model 2

S1.2 CXIDB17: Early-generation CSPAD data 2

S1.3 CXIDB76: Rayonix MX170-XFEL data 3

S1.4 CXIDB164: PILATUS 6M data 3

S2 Supplemental Figures **4**

Description of results

Unless otherwise specified, the reported *Resonet* resolution and overlapping lattice predictions are average predictions across the four quadrants in each image. Spotfinding and indexing were carried out using *DIALS*. For indexing, we did not use multiple-lattice settings, so if the data contained diffraction from multiple lattices, the indexing solution only aligned to one of the lattices. Therefore, for images which contain overlapping lattice diffraction, we expect the "fraction of Bragg spots that are indexed" to be smaller on average than

what is observed for single-lattice images. The code and meta-data needed to reproduce most of these results is available at (https://github.com/dermen/resonet/tree/master/cxidb), or can be obtained by emailing D Mendez (dermen@slac.stanford.edu).

S1 Processing serial crystallography data from CXIDB

In an attempt to explore the generality of our current *Resonet* models (trained on EIGER 16M, PILATUS 6M, and Rayonix MX-340 XFEL images) we applied them to various datasets from the Coherent X-ray Imaging Database (CXIDB, [1]). For this task, we selected CXIDB17, CXIDB76 (both XFEL serial crystallography), and CXIDB164 (synchrotron serial crystallography). For CXIDB17 and CXIDB76 datasets, our original *Resonet* resolution mode*Resonet*l overestimated the resolution, as detailed in the next sections. We assumed this was largely due to the CXIDB data being collected at close detector distances ranging from 60 - 103 mm, and relatively longer wavelengths of 1.3 Å, whereas our models were trained on simulated images where the detector distances ranged from 200 - 300 mm, and the X-ray wavelength was fixed at 0.9795Å. So, for testing those XFEL datasets we retrained the resolution model with different wavelengths and detector distances as outlined in Section S1.1.

S1.1 Retraining the *Resonet* resolution model

We generated a synthetic dataset composed of 50,000 PILATUS 6M images and 50,000 EIGER 16M images. The images were similar to those used to train the original *Resonet* resolution model (e.g., main text Figure 2), however for each of these new images, the photon wavelength was a uniform random number between 1.298 - 1.326 Å, and the sample-to-detector distance was randomly chosen as either 60 mm, 80 mm, 93 mm, or 106 mm (the distances used in the CXIDB experiments). On a single NVIDIA A100 GPU, each PILATUS 6M, EIGER 16M image took 1.46, 5.6 seconds to simulate (on average), respectively. Training the model took 75.1 seconds per epoch using 16 A100 GPUs in parallel on the NERSC Perlmutter cluster, and we trained it for 61 epochs (for comparison, a single A100 completed an epoch in 10.7 minutes).

S1.2 CXIDB17: Early-generation CSPAD data

CXIDB17 provides 138,808 raw CSPAD measurements of lysozyme diffraction. The CSPAD consists of 64 small ASICS (185 x 194 pixels each) which assemble into an area of roughly 1700 x 1700 pixels (see Figure S1). It is widely known that the early CSPAD data were contaminated by detector artifacts, especially gain non-uniformity (see for example the Supplemental Information of [2]). The *Resonet* models we trained expect 512 x 512 quadrant images, and because the assembled CSPAD format is smaller than the formats we used

for training (EIGER 16M, PILATUS 6M, Rayonix MX-340), we first had to resize the reconstructed CSPAD images to 2048 x 2048 pixels (using a bicubic interpolation). Then, we applied our 2 x 2 downsampling scheme defined in main text section 2.2 (see Figure S2). The reported resolution limit from these data was 1.9 Å [3]. As shown in Figure S3, our original *Resonet* model over-estimated the resolution, and we suspected this was because the synthetic data used to train the model were confined to a short wavelength 0.9795 Å and detector distances ranging from 200 - 300 mm, whereas the CXIDB17 CSPAD data was collected using 1.32 Å photons and a 93 mm detector distance. When applying the retrained *Resonet* model (detailed in Section S1.1) to these data, we saw a much better agreement (Figure S3) with the reported resolution.

Regarding *Resonet* overlapping lattice prediction, we found that images whose predictions $p_i \geq 0.5$ (meaning they likely contained diffraction from overlapping lattices) had fewer of their observed Bragg peaks indexed (see Figure S3).

S1.3 CXIDB76: Rayonix MX170-XFEL data

We tested *Resonet* models on three Rayonix MX170-XFEL datasets from CXIDB76 that were originally used in [4] to develop a neural network "hit finder", i.e., a model that could predict the presence of Bragg diffraction in images without explicitly performing spotfinding. The result of running *Resonet* on those images is summarized in Figures S4 to S6. For the work in [4], the images were manually labeled as "hit", "miss", or "maybe" by an expert, and we saw that those "expert annotations" agreed well with our results. Similar to CSPAD images, the Rayonix MX170-XFEL images from CXIDB76 were small in size (1920 x 1920 pixels) and in order to extract 512 x 512 pixel quadrants, we first resampled the images to 2048 x 2048 pixels before applying a 2 x 2 maxpool downsampling. For the results shown in Figures S4 to S6 we used the retrained *Resonet* resolution model described in Section S1.1.

S1.4 CXIDB164: PILATUS 6M data

This serial crystallography dataset collected at the Pohang Light Source-II synchrotron facility was very similar in diffraction geometry to the MX data we reported on in the main text, as the data were collected at 0.9796 Å and the sample-to-detector distance was 298 mm. Therefore, our original *Resonet* model was expected to perform well on them. The result of processing the data is shown in Figure S7, indicating good performance.

S2 Supplemental Figures

Figure S1: A CSPAD image from CXIDB17 showing diffraction and detector artifacts.

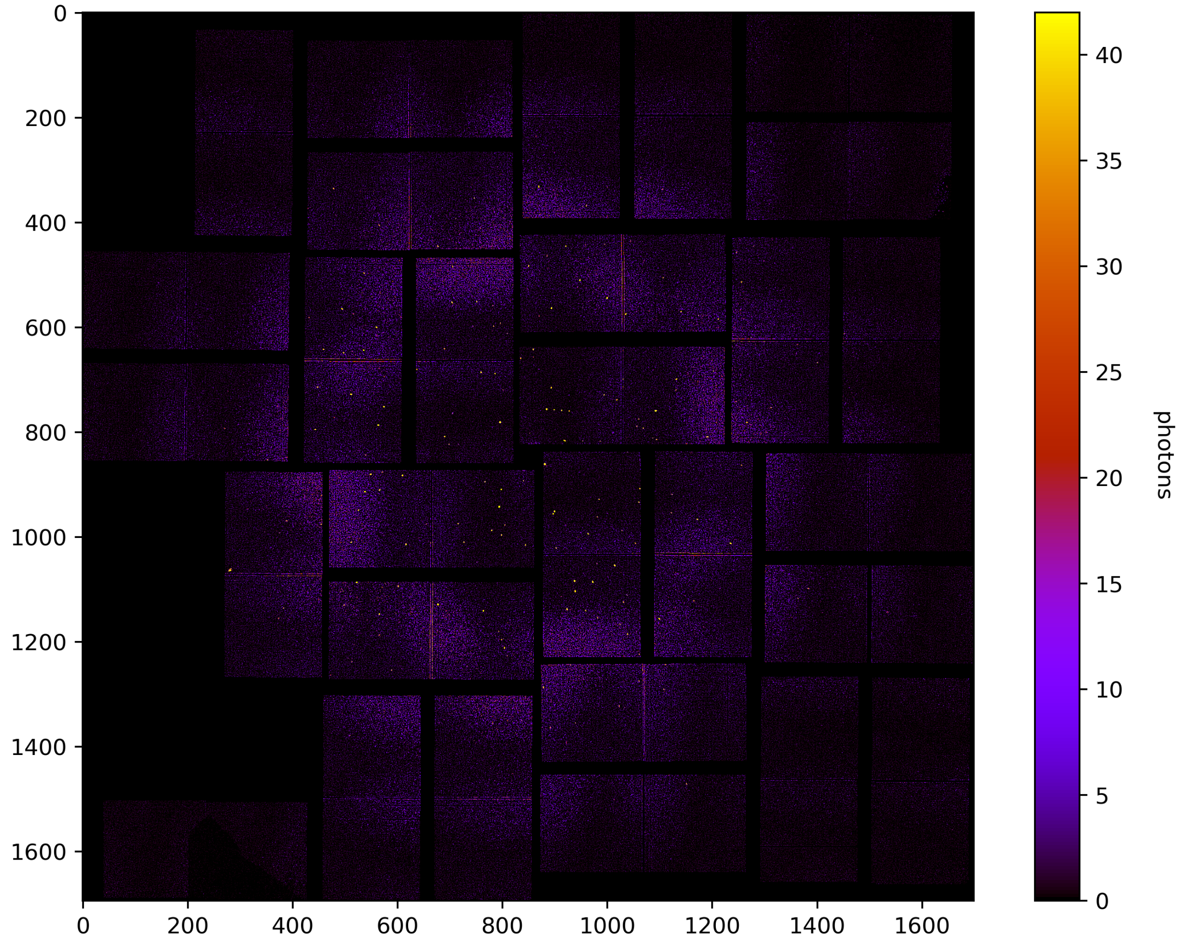

Figure S2: A CSPAD quadrant after it was conditioned for *Resonet* (see section 2.2 of main text). The white regions represent the bad pixel mask provided in the CXIDB17 deposition which we used for this analysis.

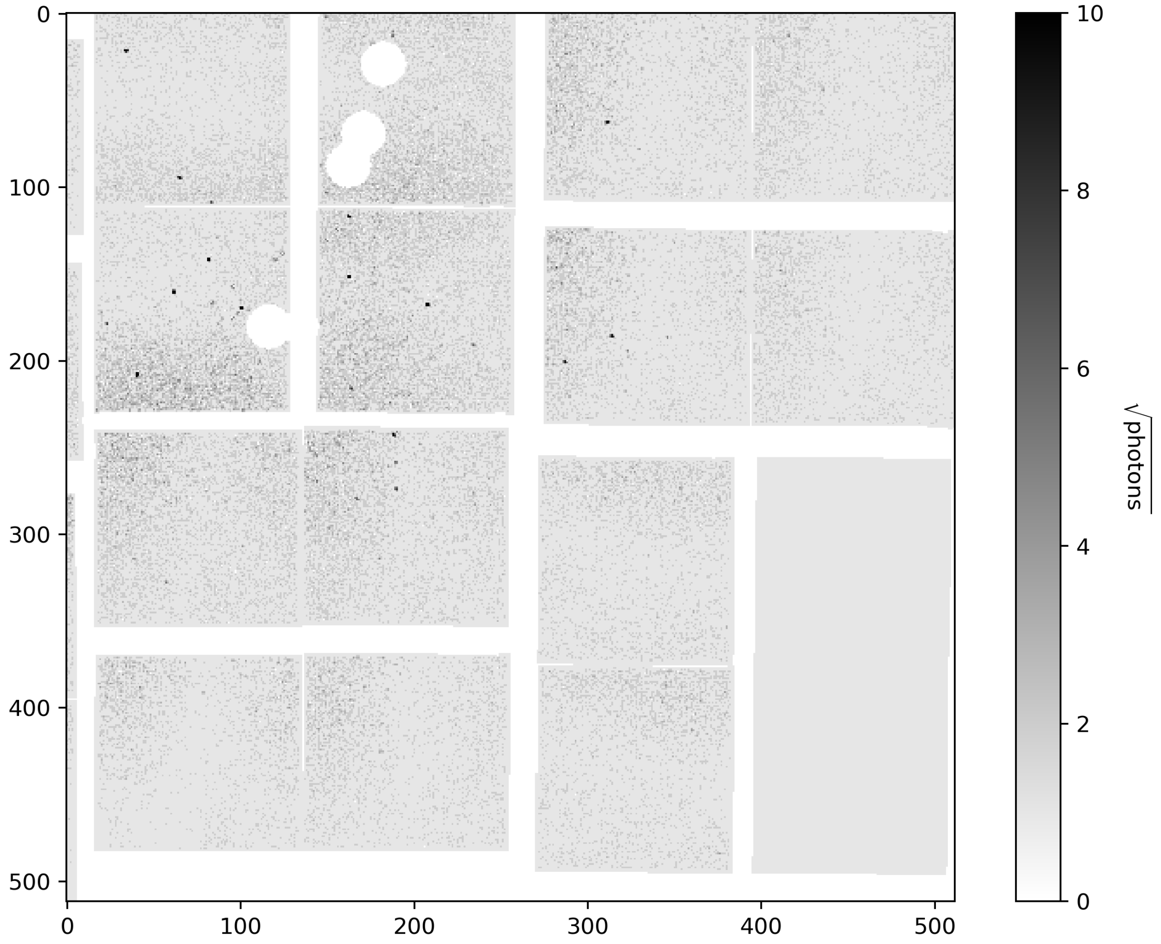

Figure S3: *Resonet* resolution and overlapping lattice predictions for CSPAD data of lysozyme from CXIDB17. (Left) *Resonet* resolution for all 138,808 images. The vertical dashed line marks 1.9 Å, the resolution of these data published in [3]. Before retraining, the model over-estimated the resolution. After retraining as described in Section S1.1, the model performed better. The horizontal axis is truncated at 4.1 Å for clarity; beyond this, resolution estimates (including for blank images) continue to decay towards 20 Å. We note that the corner resolution for this experiment was ≈ 1.5Å, yet for a small percentage of images, the predicted resolutions were below this value. Though part of this discrepancy likely results from *Resonet* inaccuracy, we stress that *Resonet* predicts an overall B-factor, which is then converted to a resolution estimate (see main text section 2.1.1 and main text Figure 1). Therefore, it is conceivable that a predicted B-factor might correspond to a resolution estimate beyond the detector corners. For future work, we will aim to provide confidence measures on every prediction, or flag predictions that are unphysical given the experimental geometry. (Right) *Resonet* overlapping lattice prediction for 35,582 images which were indexed by *DIALS*. The vertical axis is the fraction of the strong spots that were indexed. For overlapping lattice images ($p_i > 0.5$), the fraction of strong spots indexed was lower on average.

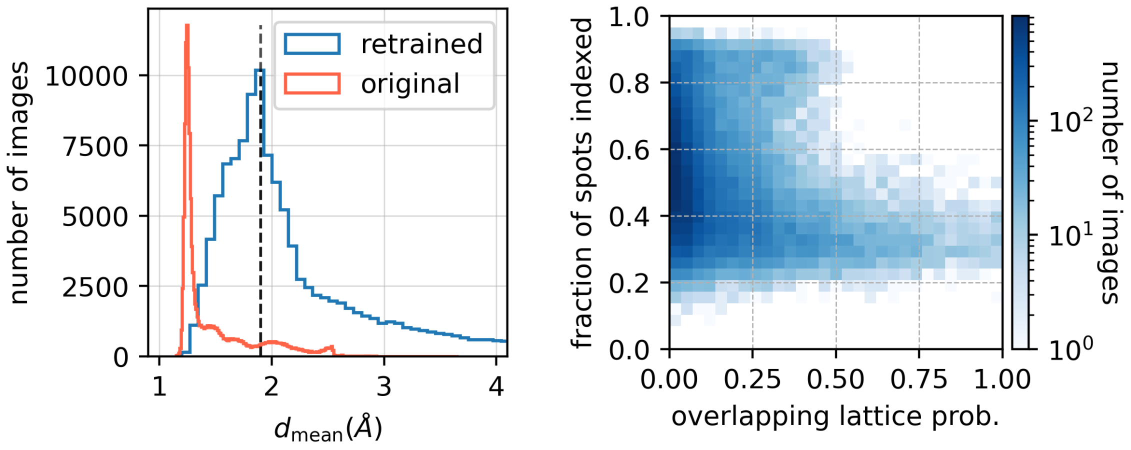

Figure S4: *Resonet* predictions for the run 95 data from CXIDB76. The data are of photosystem II ($a = 118$ Å, $b = 223$ Å, $c = 311$ Å), and collected using a drop-on-demand setup. The “hit”, “miss”, and “maybe” annotations were made available by the authors of [4] at https://github.com/nksauter/fv5080.git.

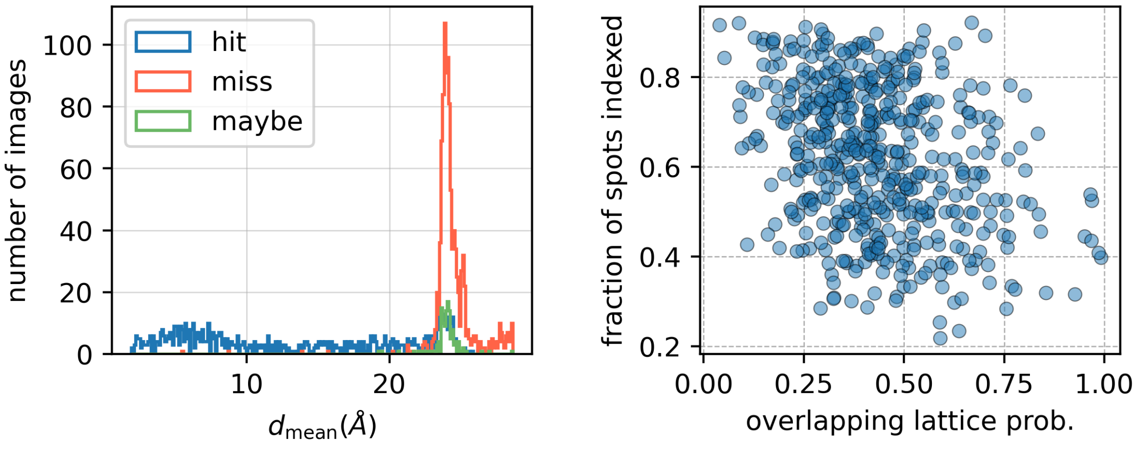

Figure S5: *Resonet* predictions for the run 18 data from CXIDB76. The data are of hydrogenase ($a = 73$ Å, $b = 96$ Å, $c = 119$ Å), and collected using a drop-on-demand setup. The "hit", "miss", and "maybe" annotations were made available by the authors of [4] at https://github.com/nksauter/fv5080.git.

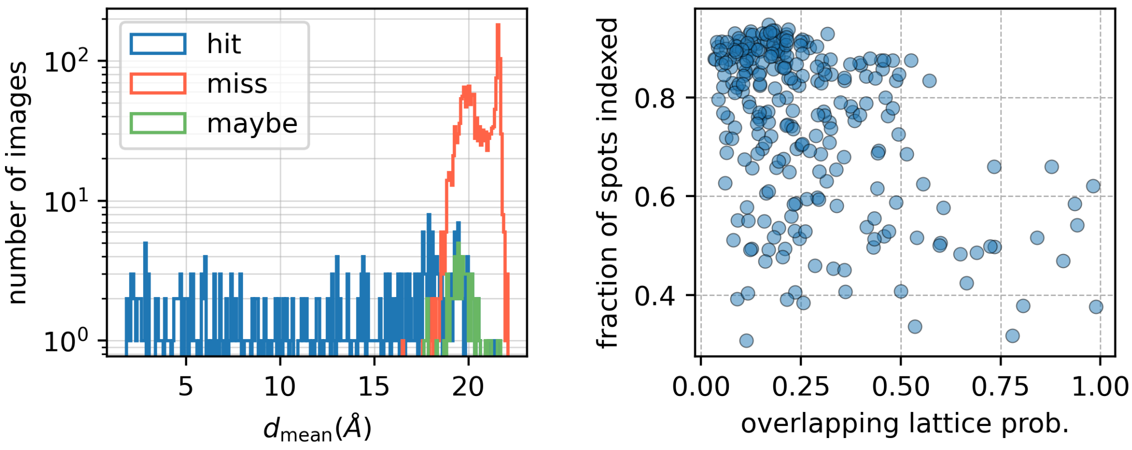

Figure S6: *Resonet* predictions for the run 20 data from CXIDB76. The data are of cyclophilin A ($a = 42$ Å, $b = 52$ Å, $c = 88$ Å), and collected using a liquid jet setup. The "hit", "miss", and "maybe" annotations were made available by the authors of [4] at https://github.com/nksauter/fv5080.git.

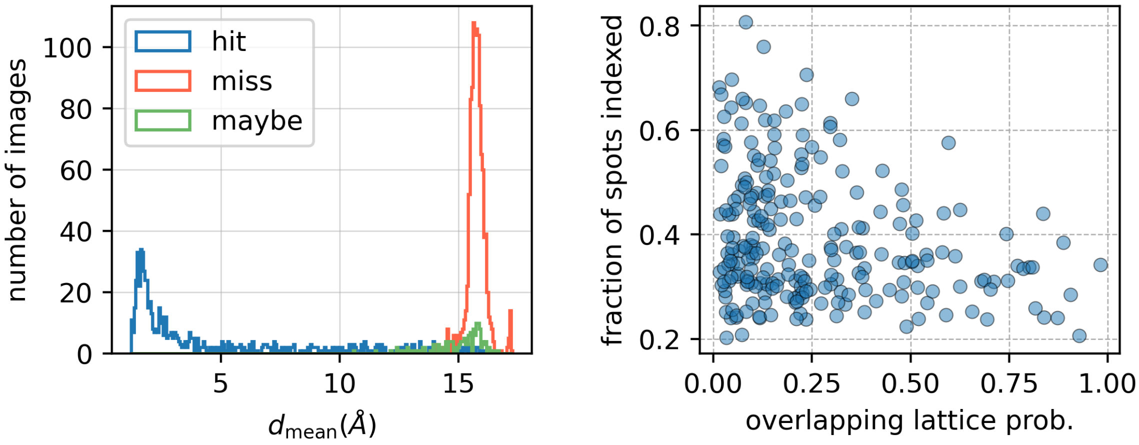

Figure S7: *Resonet* predictions for the 15,789 PILATUS 6M measurements of lysozyme in CXIDB164. The data were collected using a microfluidic chip. (Left) *Resonet* resolution estimates for the data. The vertical dashed line is 1.85 Å, the resolution published for these data in [5]. (Right) *Resonet* overlapping lattice predictions for 5,419 indexed lattices. These images all had overlapping lattice probabilities $p_i < 0.5$, however a few of the images were borderline and those images exhibited a relatively low fraction of indexed strong spots, as one should expect.

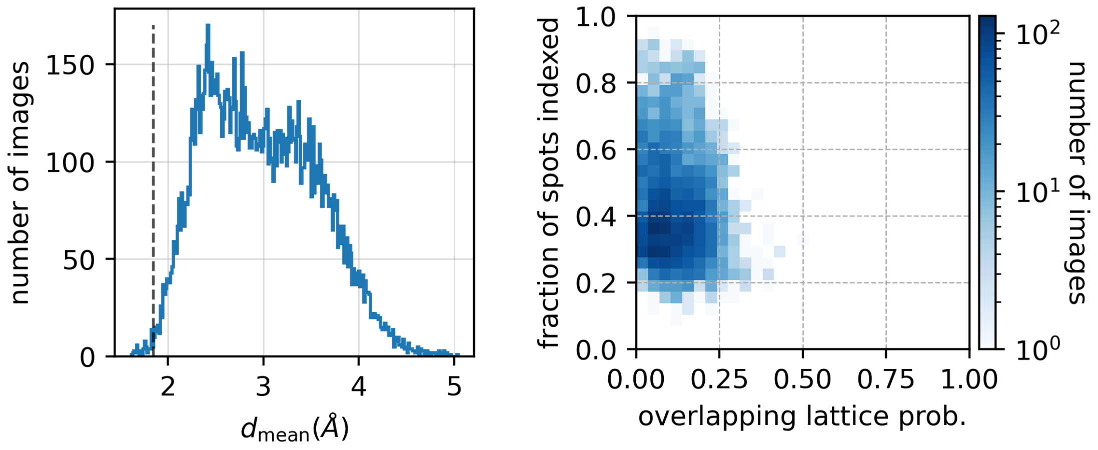

Figure S8: Timing tests for *Resonet* compared to conventional algorithms for the CXIDB164 PILATUS 6M data (5,419 indexable patterns). We tested 8-process,16-process, and 24-process jobs. For each job, the images were evenly divided amongst the concurrently running processes, and each process timed the different algorithms. The times shown are averages across every image as recorded by each individual process, normalized by the number of processes (8,16, or 24). Timing for "quad downsampling" represents the net time to convert an image into 4 quadrants, and timing for "*Resonet* resolution" and "*Resonet* overlapping lattice" represents the net time to convert all four 512 x 512 quadrants into either resolution estimates or overlapping lattice probabilities. We ran these tests on a single Perlmutter node, but only utilized one A100 GPU, such that for the *Resonet* algorithms (downsampling and inference), all processes shared the GPU. As far as we know, resolution prediction and detecting multiple lattices with conventional tools requires at least determining the strong spots and sometimes indexing them. For example, to determine resolution for individual images, *DIALS* analyzes the strong spots and their intensities, whereas e.g., *CrystFEL* looks at the positions of indexed reflections [6]. And for predicting multiple lattice scattering, one can compare the number of observed Bragg peaks to the number of indexed Bragg peaks (the former should be higher for multiple lattice events). Therefore we show here a time comparison of *Resonet* data processing compared to conventional spot finding and stills indexing, both using *DIALS*.

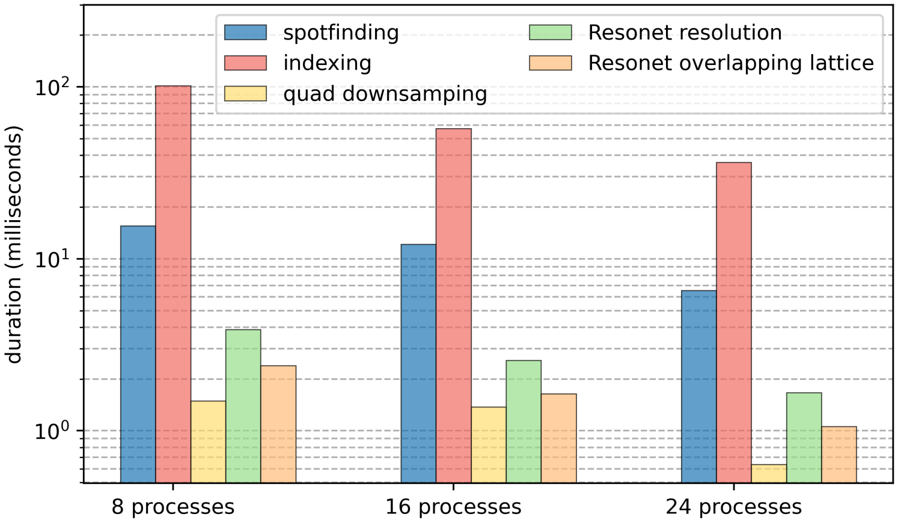

Figure S9: *Resonet* overlapping lattice prediction for the Cytochrome data described in main-text section 3.1. The data shown represent all 588 indexed images from a single run of data collection (the data were 11 runs in total).

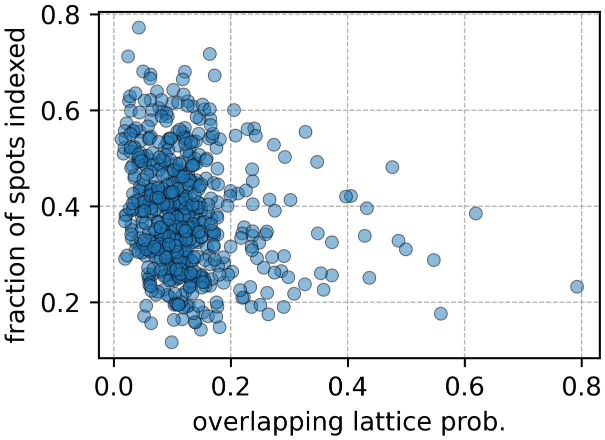

Figure S10: *Resonet* resolution estimation for the data discussed in main-text section 3.3. The data were of cracked hydrogenase crystals. The mean *Resonet* resolution for these data was 1.35 Å, whereas the published resolution for these data in [7] was 1.55 Å (indicated by the vertical dashed line). The published result only utilized 122 of the collected images which might explain the discrepancy.

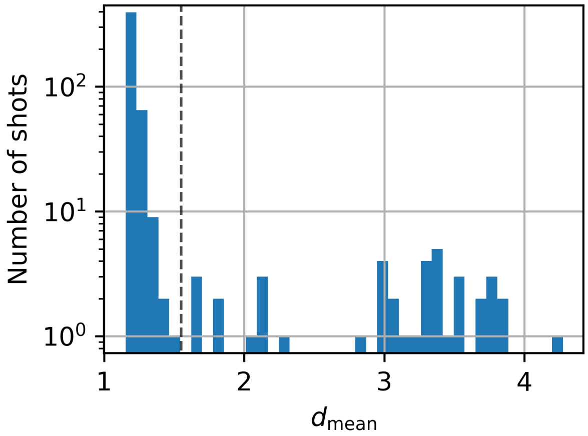

References

[1] Filipe R N C Maia. The coherent x-ray imaging data bank. *Nature Methods*, 9(9):854–855, 2012.

[2] Jonas A Sellberg, C Huang, Trevor A McQueen, ND Loh, Hartawan Laksmono, Daniel Schlesinger, RG Sierra, Dennis Nordlund, CY Hampton, Dmitri Starodub, et al. Ultrafast x-ray probing of water structure below the homogeneous ice nucleation temperature. *Nature*, 510(7505):381–384, 2014.

[3] Sébastien Boutet, Lukas Lomb, Garth J Williams, Thomas RM Barends, Andrew Aquila, R Bruce Doak, Uwe Weierstall, Daniel P DePonte, Jan Steinbrener, Robert L Shoeman, et al. High-resolution protein structure determination by serial femtosecond crystallography. *Science*, 337(6092):362–364, 2012.

[4] T-W Ke, Aaron S Brewster, Stella X Yu, Daniela Ushizima, Chao Yang, and Nicholas K Sauter. A convolutional neural network-based screening tool for x-ray serial crystallography. *Journal of synchrotron radiation*, 25(3):655–670, 2018.

[5] Ki Hyun Nam and Yunje Cho. Stable sample delivery in a viscous medium via a polyimide-based single-channel microfluidic chip for serial crystallography. *Journal of Applied Crystallography*, 54(4):1081–1087, 2021.

[6] Thomas A White, Valerio Mariani, Wolfgang Brehm, Oleksandr Yefanov, Anton Barty, Kenneth R Beyerlein, Fedor Chervinskii, Lorenzo Galli, Cornelius Gati, Takanori Nakane, et al. Recent developments in crystfel. *Journal of applied crystallography*, 49(2):680–689, 2016.

[7] Jacob H. Artz, Oleg A. Zadvornyy, David W. Mulder, Stephen M. Keable, Aina E. Cohen, Michael W. Ratzloff, S. Garrett Williams, Bojana Ginovska, Neeraj Kumar, Jinhu Song, Scott E. McPhillips, Catherine M. Davidson, Artem Y. Lyubimov, Natasha Pence, Gerrit J. Schut, Anne K. Jones, S. Michael Soltis, Michael W. W. Adams, Simone Raugei, Paul W. King, and John W. Peters. Tuning catalytic bias of hydrogen gas producing hydrogenases. *Journal of the American Chemical Society*, 142(3):1227–1235, 2020.
